# Computational mechanisms of perception in autism revealed using games inspired by rodent operant tasks

**DOI:** 10.1126/sciadv.aec9291

**Published:** 2026-09-11

**Authors:** Sucheta Chakravarty, Quan Do, Yutong Li, Vanessa Torres-Lacarra, Helen Tager-Flusberg, Joseph T. McGuire, Benjamin B. Scott

**Affiliations:** ^1^ Psychological & Brain Sciences, Boston University, Boston, MA, USA.; ^2^ Graduate Program for Neuroscience, Boston University, Boston, MA, USA.; ^3^ Center for Systems Neuroscience, Boston University, Boston, MA, USA.

## Abstract

Altered perception is a hallmark of autism spectrum disorder (ASD), yet its underlying mechanisms remain unclear, in part because individuals with greater impairments are often excluded from psychophysics research. Here, we introduce GEODE (gathering evidence to optimize decisions), an online video game designed to measure visual perception across the full autism spectrum. GEODE incorporates feedback-based shaping from animal training to teach task mechanics, customized graphics and storyline elements to sustain engagement, and touchscreen compatibility for broader accessibility, enabling participation from adolescents including those with profound autism. Across a large, heterogeneous cohort, autistic participants successfully played GEODE but showed slower learning and reduced accuracy compared with typically developing siblings. These deficits were best explained by increased noise in the integration of sensory evidence, which scaled nonlinearly with stimulus complexity and tracked standardized survey measures of adaptive functioning more closely than diagnostic category alone. Injecting equivalent noise into artificial neural networks produced agents that recapitulated ASD-like patterns of learning and decision-making. These findings implicate noisy evidence integration as a computational mechanism for altered visual perception across the autism spectrum and establish a scalable, accessible platform for probing altered perception and learning in neurodevelopmental and neuropsychiatric disorders.

## INTRODUCTION

Atypical perceptual processing is considered a core feature of autism spectrum disorder (ASD) (*1*–*3*). However, the exact nature of perceptual alteration remains unclear (*4*–*6*). Psychophysical studies indicate that individuals with ASD perform similarly to typically developing (TD) controls on tests of simple detection or discrimination but show deficits on more complex tasks requiring the integration of sensory evidence over time (*3*, *7*, *8*). These findings have fueled the hypothesis that atypical perception in ASD reflects impairments in perceptual integration, a critical process when stimuli are noisy or temporally extended, such as in speech (*9*, *10*).

Several mechanistic models have been proposed to account for deficits in perceptual integration in ASD. Slower response times may reflect elevated decision boundaries (*11*) or reduced evidence accumulation rates (*8*), whereas greater within-subject variability in stimulus-evoked responses suggest increased noise in the perceptual process (*12*, *13*). However, it remains unclear whether altered perception in ASD arises from bottom-up sensory encoding, top-down control, or multiple mechanisms that vary across individuals (*6*, *14*–*17*). Crucially, how such perceptual differences relate to other clinically relevant autism traits remains unknown (*5*).

Progress in resolving these questions has been slowed by methodological constraints. Previous psychophysical studies have largely focused on small cohorts, involving ASD populations matched with nonautistic groups, whereas individuals with more limited language and intellectual ability are often excluded. This exclusion has long been recognized as a major problem in ASD research (*18*–*20*). Individuals with severe symptoms are more challenging to study, yet they have the greatest clinical need and stand to benefit most from improved understanding (*21*, *22*). In addition, animal models, where circuit mechanisms can be addressed, mimic more severe forms of ASD (*23*). This reliance on narrow samples may contribute to inconsistent results and limits broader mechanistic understanding of perception in ASD (*7*, *8*, *24*–*27*).

To address these challenges, we developed GEODE (gathering evidence to optimize decisions), an online video game designed to measure perceptual integration across the autism spectrum. GEODE adapts pulse-based tasks originally developed in rodents to dissect alternative cognitive models of evidence accumulation (*28*, *29*) and incorporates feedback-based shaping procedures from animal training, allowing participants to learn the task without verbal instructions. We introduced key upgrades tailored to adolescents with ASD: enhanced graphics, a motivating multilevel storyline, and touchscreen compatibility. Combined with online deployment for remote testing, these features were designed to maximize accessibility and engagement, making the paradigm usable by individuals with limited communication or profound autism.

Using GEODE, we collected gameplay data from a large, heterogeneous cohort of adolescents with ASD and their TD siblings. On average, autistic participants exhibited slower learning and reduced accuracy, with deficits consistent with greater noise in perceptual integration. Across individuals, computational modeling revealed that noise levels tracked the severity of clinically relevant symptoms, correlating most strongly with adaptive functioning. Last, injecting equivalent noise into artificial agents reproduced ASD-like patterns of learning and decision-making. Together, these data support a model in which perceptual and learning deficits in ASD arise, in part, from increased noise in sensory integration. Moreover, these results demonstrate the value of online video games with nonverbal training pipelines for efficient, high-throughput assessment of perceptual and cognitive functioning across ASD.

## RESULTS

### GEODE game design and accessibility upgrades

Our goal was to develop a platform capable of measuring visual perception across the ASD spectrum. To overcome existing obstacles, an ideal paradigm should satisfy several key criteria. First, it must be scalable, capable of generating large datasets that provide the quantitative rigor needed to characterize sensory processing (*5*). Second, it should be sufficiently complex to reveal meaningful differences in perceptual integration, yet simple and engaging enough to be playable across the full spectrum, including individuals with profound autism. Third, it should be accessible from a participant’s home to maximize inclusivity and participation. Last, to advance translational research, the paradigm should be compatible with animal models, enabling cross-species studies that connect behavioral phenotypes to underlying cellular and circuit mechanisms (*30*, *31*).

**Fig. 1.**
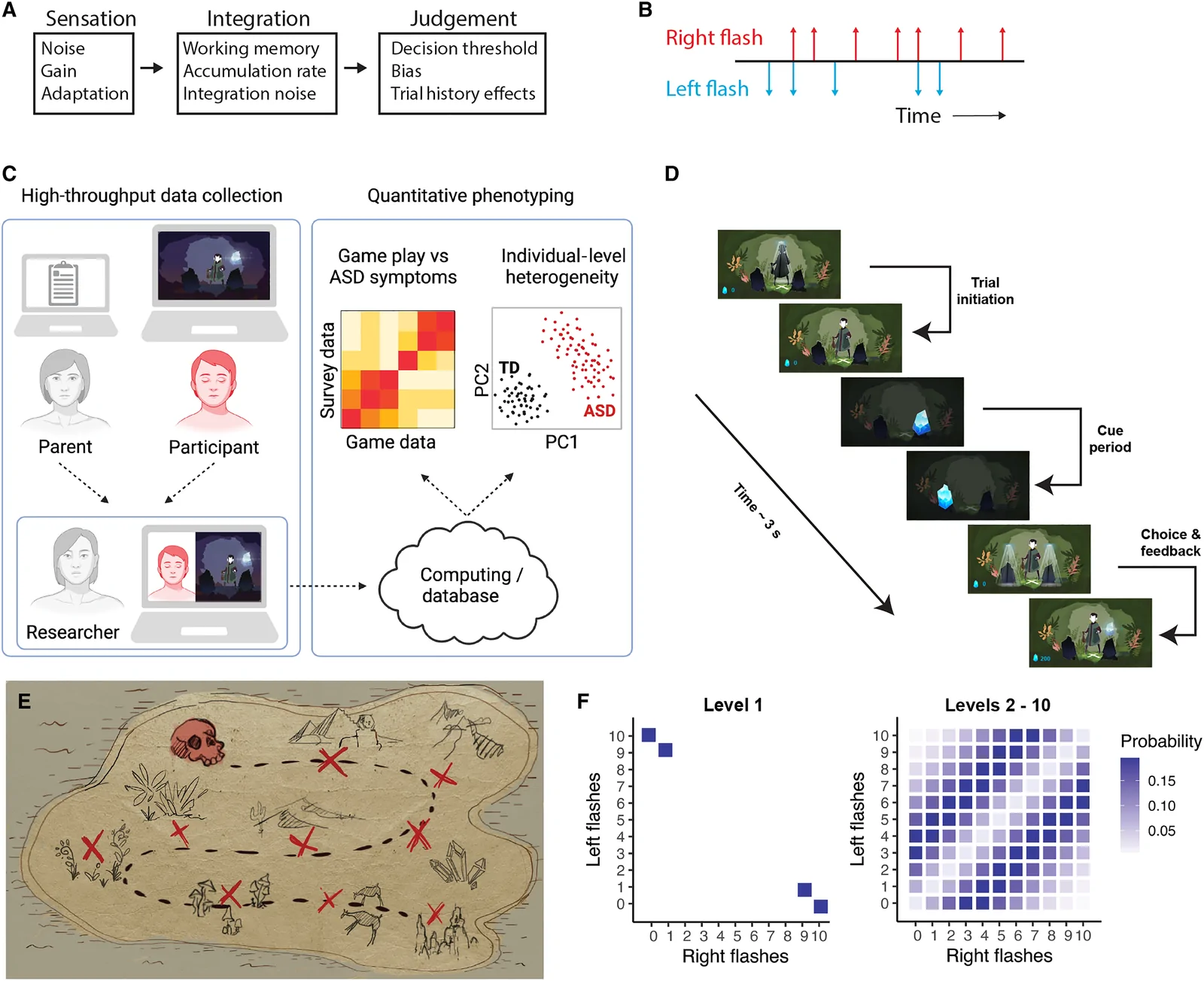
Overview of GEODE. (**A**) Atypical perceptual processing in ASD may be due to deficits in early sensory input or later processing stages, and the exact nature of which remains to be understood. (**B**) GEODE used brief light flashes presented to the left and right hemifields, at random time points. Participants were rewarded for choosing the side (left/right) with more flashes. (**C**) GEODE leverages high-throughput online data collection to enable quantitative phenotyping and data-driven analysis of heterogeneous ASD cohorts. (**D**) Trials were initiated by clicking on avatar in the screen center; flashes (in the shape of gems) appeared during the cue period. After the cue period, the participant had to make a side choice and click on it; only correct choices (to the side with more total flashes) were rewarded with points, which accumulated over trials. (**E**) A treasure map informed players about game progress through 10 levels (indicated by “X” and a skull). (**F**) The choice rule was not given verbally; participants learned through trial and error. For the first 10 trials for training (level 1), the numbers of flashes on the two sides were 10 versus 0 or 9 versus 1. For the remaining 190 trials (levels 2 to 10), the flash ratio between the two sides averaged at 70:30 whereas the precise flash times and numbers were generated stochastically. Panel (B) Created in BioRender. Scott, B. (2026) https://BioRender.com/5mi76oo.

To achieve these goals, we developed GEODE a video game inspired by pulse-based psychophysics tasks originally designed for rodents (Fig. 1). In these tasks, which were designed to isolate the contribution of distinct cognitive processes to perceptual judgements (Fig. 1A), participants are presented with brief light pulses (flashes) to the left and right visual hemifields and rewarded for choosing the side with more flashes (Fig. 1B). The discrete nature of the pulses provides precise experimental control over the accumulation process: The number, duration, and timing of pulses can be varied per trial to yield a rich dataset for model-based analysis of perceptual decisions.

An earlier version of this game was validated in human adults (*32*). GEODE uses a similar feedback-based training pipeline modeled on shaping procedures from animal studies, enabling participants to learn the rules without verbal instruction. Here, we upgraded the platform specifically for adolescents with ASD and related neurodevelopmental disorders (NDDs). First, we enhanced the graphics to increase engagement. Second, we embedded the task in a storyline with multiple levels displayed on a map to sustain motivation. Third, we added touchscreen functionality in addition to mouse and keyboard input to broaden accessibility. Together, these modifications ensured that the game was usable and engaging for a wider range of participants, including those with limited communication abilities.

From the player’s perspective, the game presented a quest to collect gems (Fig. 1D). Each trial began when the player clicked an avatar at the center of the screen (initiation), after which gemlike flashes appeared bilaterally (cue period). The player then made a choice, receiving points for correct responses that accumulated across trials. Progress was shown on a treasure map with 10 levels (Fig. 1E). Training began with 10 easy discrimination trials (10 versus 0 or 9 versus 1 flashes). After training, the full stimulus set was introduced, with flashes generated stochastically on each trial (*P* = 0.7 correct side, *P* = 0.3 incorrect side; *n* = 190 trials; Fig. 1F).

### Participant recruitment and characterization with standardized surveys

Participants were recruited from several databases, including SPARK, Simons Searchlight, the Phelan-McDermid Syndrome Foundation (PMSF) data hub, and the Boston University Center for Autism Research Excellence (CARE) registry. In total, *N* = 308 adolescents (ages 11 to 17; 198 male, 90 female, and 20 unidentified) played GEODE, comprising *N* = 212 with a diagnosis of ASD and *N* = 96 TD siblings (fig. S1).

To further characterize participants, a subset of parents or guardians completed four ASD-relevant surveys online: the Social Responsiveness Scale (SRS-2) (*33*), Vineland Adaptive Behavior Scales (VABS-3) (*34*), Adult/Adolescent Sensory Profile (AASP) (*35*), and Behavioral Inflexibility Scale (BIS) (*36*). The SRS-2 provided an index of ASD symptom severity. The VABS-3 (reported for *N* = 85 TD and *N* = 195 ASD) assessed adaptive functioning; a threshold score of 75 distinguished participants functioning at a higher level (≥75) from those with lower-adaptive behavior and greater support needs (<75), which often co-occur with intellectual disability.

**Fig. 2.**
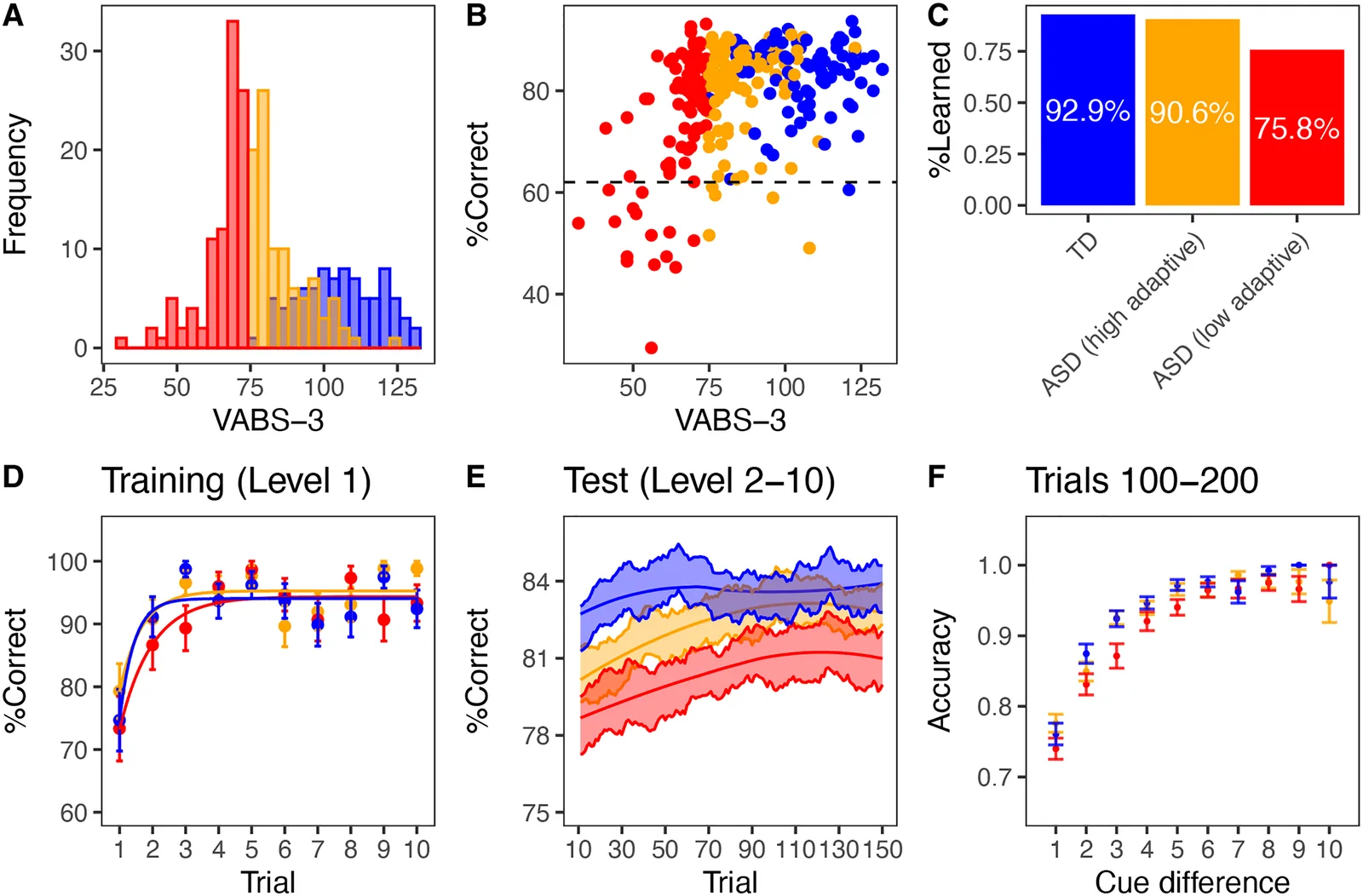
Participants across the ASD spectrum learned GEODE. (**A**) We recruited 218 adolescents with ASD and 99 TD siblings. ASD varied in symptom severity, reflected in their VABS-3 score (absolute composite). We categorized participants into three groups: TD, ASD high adaptive (with VABS-3 standard scores 75 or higher), and ASD low adaptive (with VABS-3 lower than 75). (**B**) Most participants in each group achieved an accuracy rate of 0.62 (upper limit of 99.9% binomial CI for chance performance) or above. (**C**) Most participants passed task criteria (complete all 200 trials and average accuracy over 62%). (**D**) Participants who passed criteria performed similarly across groups during training (level 1). (**E**) The three groups showed differences in learning rate and overall performance during test trials (levels 2 to 10). ASD participants showed slower learning compared to TD participants, and ASD participants with low-adaptive behavior scores differed even more. Plot shows rolling averages of accuracy over 50-trial bins. (**F**) Psychometric function plots the proportion of correct choices as a function of the flash or cue (absolute) difference. Error bars represent binomial CIs.

The ASD sample spanned the spectrum, as reflected in the range of SRS-2 and VABS-3 scores (Fig. 2, A and B, and fig. S2), and included a small number of individuals with Phelan-McDermid syndrome (*N* = 3) and 16p deletion syndrome (*N* = 3). Over 90% of TD participants (*N* = 79/85) and ASD participants with higher-adaptive behavior scores (*N* = 87/96, VABS-3 ≥ 75) met criteria for successful gameplay (200 trials with ≥61.6% accuracy). More than 75% of ASD participants with lower-adaptive behavior scores (*N* = 75/99, VABS-3 < 75) also achieved success, including some with VABS-3 scores below 50, in the profound autism range (*21*).

### Individuals across the ASD spectrum learned GEODE successfully

We next asked how participants across this heterogeneous cohort learned and engaged with GEODE. Analysis of gameplay revealed that ASD and TD participants rapidly acquired the basic rules within a few trials, with no significant group differences in pairwise comparisons during the initial training phase (trials 1 to 10; Fig. 2D). After transition to the full stimulus set, accuracy declined abruptly before gradually recovering to a plateau (Fig. 2E). At plateau, TD as well as high- and low-adaptive ASD players were sensitive to the difference in the number of flashes on each side (Fig. 2F) .

Quantitative analysis of the learning curves revealed significantly lower initial accuracy, slower learning rates, and reduced plateau performance for high- and low-adaptive ASD participants compared with TD participants [bootstrap learning curve fits with Kolmogorov-Smirnov (KS) tests for *A*_0_, *L*, and *P*; all pairwise comparisons *P* < 2.2 × 10^−16^]. Additional analyses showed greater heterogeneity in early-learning dynamics within ASD (Kruskal-Wallis, *P* = 0.00033), with early-learning slopes predicting performance at the transition to the test trials (*P* = 0.002; see Materials and Methods). These data indicate that adolescents across the autism spectrum were able to successfully learn and play GEODE but that, on average, they exhibited slower learning and slightly lower accuracy compared to their TD peers.

### Individuals with ASD showed deficits in evidence accumulation

**Fig. 3.**
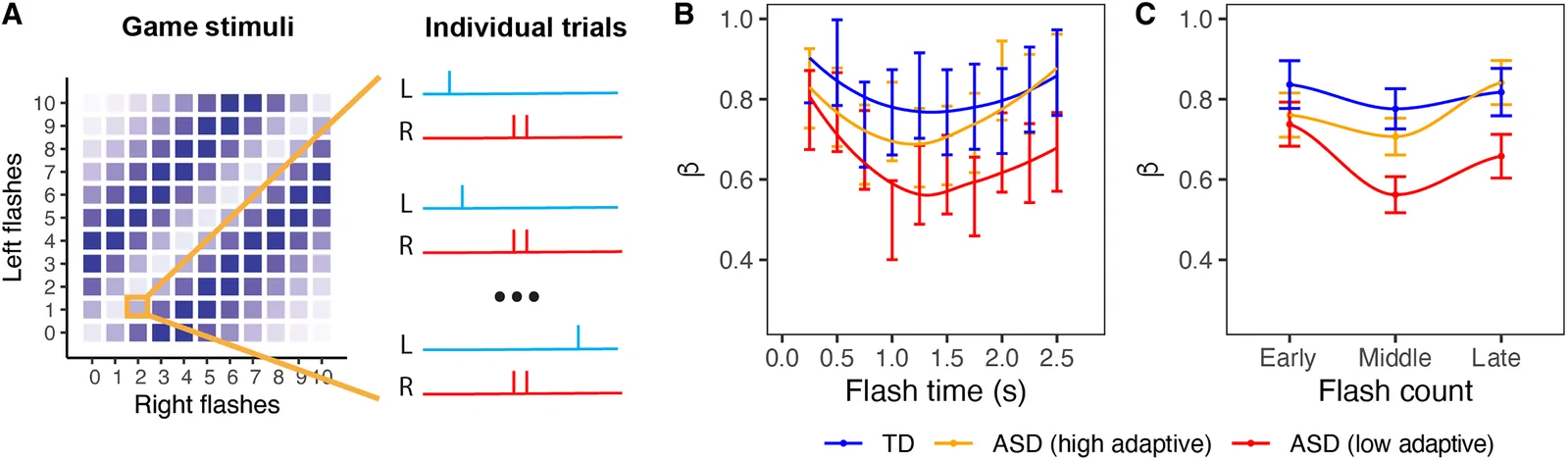
ASD participants showed deficits in evidence accumulation. (**A**) The pulse-based paradigm incorporated high-dimensional stimuli; on each trial, sensory information was presented at different time points; each flash lasted 50 ms and flashes occurred in time bins of 250 ms. Trial-by-trial stimulus details facilitate characterization of participants’ perceptual choice behavior. (**B**) A logistic regression model estimated the weight (beta) of each flash (bin) on behavioral choice for TD (blue), ASD high-adaptive (gold), and ASD low-adaptive (red) participants. (**C**) Logistic regression model for behavioral choice grouping early, middle, and late flashes together. Models used data pooled across participants, and model weights were collapsed across left and right sides; error bars are CIs of the average weight (combined for left and right), calculated using error propagation method.

We next leveraged GEODE’s precisely timed sensory pulses to probe how participants weighted sensory evidence (Fig. 3). We first asked how the timing of flashes influenced decisions in ASD and TD groups. Using logistic regression on pooled choice data (250-ms bins for left and right flashes; Fig. 3A), we found broadly similar integration time courses across groups: Evidence was accumulated stably throughout the trial, with modest recency and primacy biases. However, both ASD groups showed lower overall regression weights compared to TD (Fig. 3B). A significant main effect of group [*F*(2,24) = 13.94, *P* < 0.001, partial η^2^ = 0.54] was observed, with post hoc tests revealing differences between TD and lower-adaptive ASD participants (Cohen’s *d* = 2.29, *P* < 0.001) and between the two ASD groups (Cohen’s *d* = 1.64, *P* < 0.01). These findings were consistent when flashes were grouped into early, middle, and late bins (Fig. 3C). Thus, although all groups used a similar accumulation strategy, ASD participants used integrated sensory evidence less effectively.

We next examined how the number of flashes influenced accuracy. Three-dimensional (3D) psychometric functions revealed that ASD participants performed worse than TD participants both when the difference between sides was small and when the total number of flashes was high (fig. S3, A to C). These results raise the possibility that accuracy in ASD was limited by a noisy integration process, with noise increasing as more sensory evidence was accumulated.

### Greater integration noise explains deficits in evidence accumulation in ASD

**Fig. 4.**
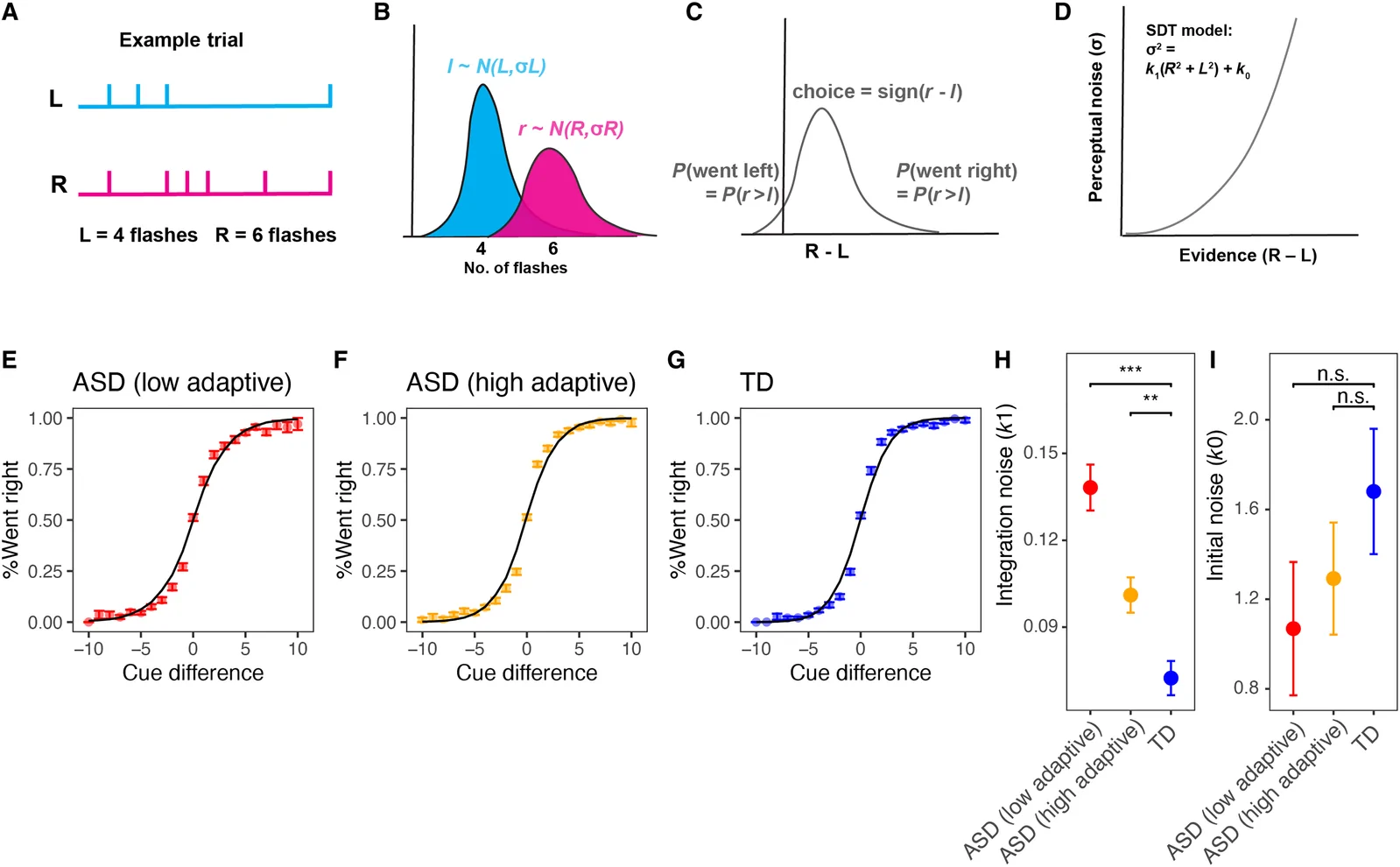
ASD participants experienced greater integration noise. (**A**) Schematic of an example trial with four flashes on the left and six flashes on the right. (**B** and **C**) An SDT model estimated flashes from Gaussian random variables, with the mean reflecting evidence strength and variance reflecting internal noise. (**D**) The model assumed scalar integration noise, associated with a free parameter (*k*1), and initial noise, associated with a free parameter (*k*0). (**E** to **G**) Model fits (solid line) versus original data (error bars) for the ASD low-adaptive (E), ASD high-adaptive (F), and TD (G) groups. (**H** and **I**) Model fits revealed greater integration noise in both ASD groups compared with TD (H) and no significant difference in initial noise across groups (I). Error bars are CIs on the parameter estimates. n.s., not significant. **P < 0.01, ***P < 0.001 (Benjamini-Hochberg adjusted).

To characterize the nature of noise in the integration process across ASD, we analyzed behavioral choices using signal detection theory (SDT). In this framework, the perception of each sequence of flashes was modeled as a real number drawn from a Gaussian distribution, reflecting uncertainty in the participant’s estimate of the number of flashes (Fig. 4, A to D). The mean of the distribution equals the true number of flashes, whereas the variance reflects subjective uncertainty.

We compared two parameterizations of uncertainty. In the linear variance model, variance scales with the number of flashes (fig. S4). In the scalar variability model, inspired by Weber-Fechner scaling, variance is proportional to the square of the number of flashes. Model comparison revealed that the scalar variability model provided a better fit for both ASD and TD participants (fig. S4).

Next, we used the better-fitting scalar variability model to estimate the magnitude of integration noise across cohorts (Fig. 4, E to G). This model included free parameters for integration noise (*k*1) and noise from all other sources (*k*0), along with terms for side bias (bs), win-stay (*b*1), and lose-switch (*b*2) (see Materials and Methods).

In pooled fits, both high-adaptive and low-adaptive ASD showed greater integration noise (*k*1) than TD [*k*1_high-adaptive ASD_ = 0.101, SE = 0.00610; *k*1_low-adaptive ASD_ = 0.138, SE = 0.00792; *k*1_TD_ = 0.0724, SE = 0.00591; Δ_high-adaptive ASD, TD_ = 0.0287, 95% confidence interval (CI): [0.0120, 0.0453], *Z* = 3.38, *P*_BH-adjusted_ = 0.00367; Δ_low-adaptive ASD, TD_ = 0.0658, 95% CI: [0.0464, 0.0852], *Z* = 6.66, *P*_BH-adjusted_ = 2.79 × 10^−10^; Fig. 4H]. No significant group-level differences in nonintegration noise (*k*0; Fig. 4I) or bias terms were observed.

### Modeling perceptual and cognitive parameters at the individual level

Next, to address how integration noise varied across the autism spectrum, we combined our SDT approach with hierarchical Bayesian modeling (HBM-SDT) (fig. S5, A and B). By leveraging group-level and individual-level data, this approach can yield reliable noise parameter estimates for individual participants with small numbers of trials (*32*). After fitting, the HBM-SDT model revealed wide variation in the parameter estimates for the ASD group as a whole (fig. S5C). Integration noise (*k*1) was significantly greater for ASD than TD [Welch two-sample test, t(252.46)=2.46,P=0.015, Cohen’s *d* = 0.276]. In addition, *k*1 correlated with psychometric slope [estimated using a generalized linear model (GLM); fig. S6], indicating correspondence between integration noise and perceptual sensitivity at the individual level.

In addition, HBM-SDT was sensitive enough to reveal significant differences in trial history parameters between TD and ASD, including greater magnitude in side bias [bs; t(227.34)=2.03,P=0.04,d=−0.075], win-stay bias [*b*1; t(233.83)=3.03,P=0.003,d=0.224], and lose-switch bias [b2;t(231.18)=2.75,P=0.006,d=0.097] for ASD compared with TD. These bias parameters capture systematic choice tendencies and sensitivity to recent outcomes, reflecting how decisions are influenced by prior choices and feedback rather than by sensory evidence magnitude alone.

Last, parameter estimates were stable across retest sessions (fig. S7). Together, the pooled SDT fits and individual level HBM-SDT fits support the idea that increased noise in the evidence integration process is a key contributor to perceptual decision-making deficits in ASD.

### Integration noise correlates with symptom severity in social and adaptive domains

**Fig. 5.**
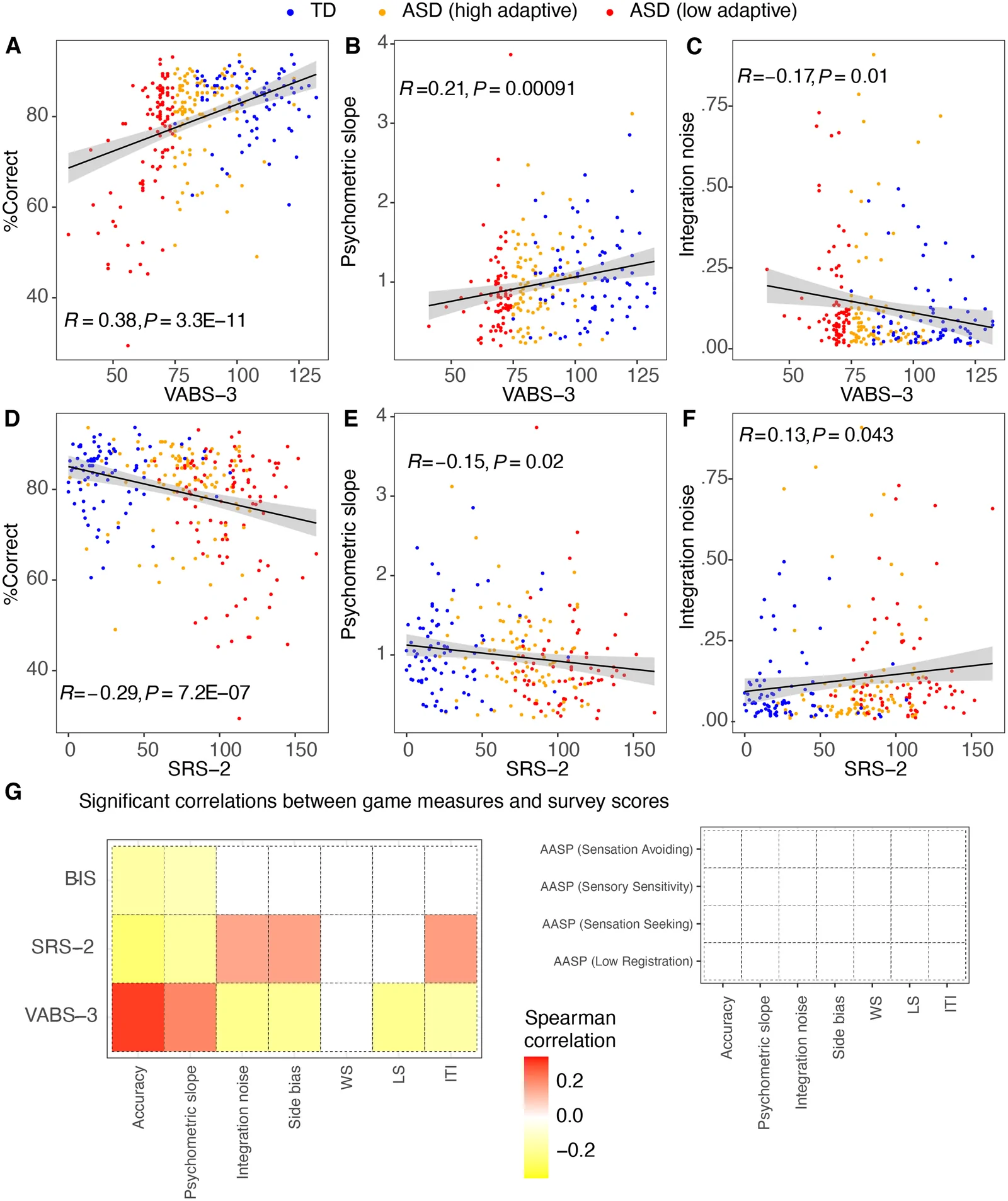
Game play correlated with symptom severity. (**A** to **F**) Correlation between game statistics and ASD symptoms from caregiver surveys. Each colored dot represents a TD (blue), ASD (orange), or low-adaptive ASD (red) individuals. Black line indicates the slope. (**G**) Correlation matrix between all computed game statistics and caregiver surveys. Color indicates the direction and strength of the correlation. White indicates no significant correlation (*P* > 0.05). No significant correlations were observed between game statistics and sensory profile scores across individuals (*P* > 0.05).

We next asked whether integration noise, and other game derived metrics, correlated with ASD symptom severity. Integration noise, and multiple other game measures, correlated most strongly with VABS-3, followed by SRS-2 (Fig. 5, A to F). Accuracy and psychometric slope also correlated with BIS scores (Fig. 5G). In contrast, no significant associations emerged with sensory profile scores (AASP). These findings highlight integration noise as an objective, quantitative marker that captures clinically relevant impairments in both adaptive and social domains.

We also examined within-group correlations. For integration noise, correlations with VABS-3 were *r* = −0.096 (*P* = 0.224) within ASD and *r* = −0.162 (*P* = 0.153) within TD. For SRS-2, correlations were *r* = 0.068 (*P* = 0.390) within ASD and *r* = 0.0047 (*P* = 0.967) within TD. The weaker within-group correlations indicate that the pooled relationships partly reflect differences between ASD and TD groups, suggesting that integration noise tracks a dimension of functional variation spanning diagnostic boundaries, rather than variation restricted to a single group.

Consistent with this interpretation, we observed no significant sex differences within TD or ASD groups in task performance, perceptual noise *k*1, VABS-3, SRS-2, or BIS (two-sided Wilcoxon rank sum tests), with the only exception being AASP sensation-seeking subscale, where ASD females showed higher scores than ASD males (*W* = 1520.5, *P* = 0.0213). Together, these results suggest that the primary task-based computational findings are unlikely to be driven by sex differences.

As a secondary analysis, we asked whether game-based measures could explain additional sources of variation among participants, beyond what is captured by the surveys. We conducted a principal components analysis (PCA) on the combined features (game and survey) from all participants with complete survey data (*N* = 243; 160 ASD). The first two PCs explained 32 and 22% of the variation in the data, respectively (fig. S8A), and the ASD and TD groups were well separated in PC space (fig. S8B). Survey measures loaded heavily onto PC1, whereas game measures loaded heavily onto PC2 (fig. S8C). Furthermore, feature scores for PC1 were significantly different for ASD and TD [Welch two-sample test, t(158.95)=−4.10,P<0.001,d=−0.56]; this was also true for PC2 [t(194.25)=2.29,P=0.02,d=0.31]. These results suggest that game-derived measures capture unique aspects of the ASD phenotype not fully reflected in traditional survey data.

To understand whether the game-based measures captured ASD-specific characteristics, we performed an additional analysis among a subsample of TD (*N* = 30) and ASD (*N* = 31) participants who match in VABS-3 scores (fig. S9, A and B). We observed no significant group differences between TD and VABS-3–matched ASD in game-derived metrics (fig. S9, C to E), whereas survey-based measures did show group differences (fig. S9, F to K). These findings could suggest that the game-derived metrics capture characteristics related to an individual’s level of impairment, such as intellectual disability, rather than diagnostic category alone. However, we note that this analysis was based on a relatively small subsample, and the effect size estimates of game-derived metrics were small with wide CIs (e.g., percentage correct: Cohen’s *d* = 0.07, 95% CI: [−0.43, 0.57]). Accordingly, the absence of group differences in game-derived metrics from these subcohorts should be interpreted cautiously in light of limited statistical power.

### Integration noise in artificial learning agents generates ASD-like traits

**Fig. 6.**
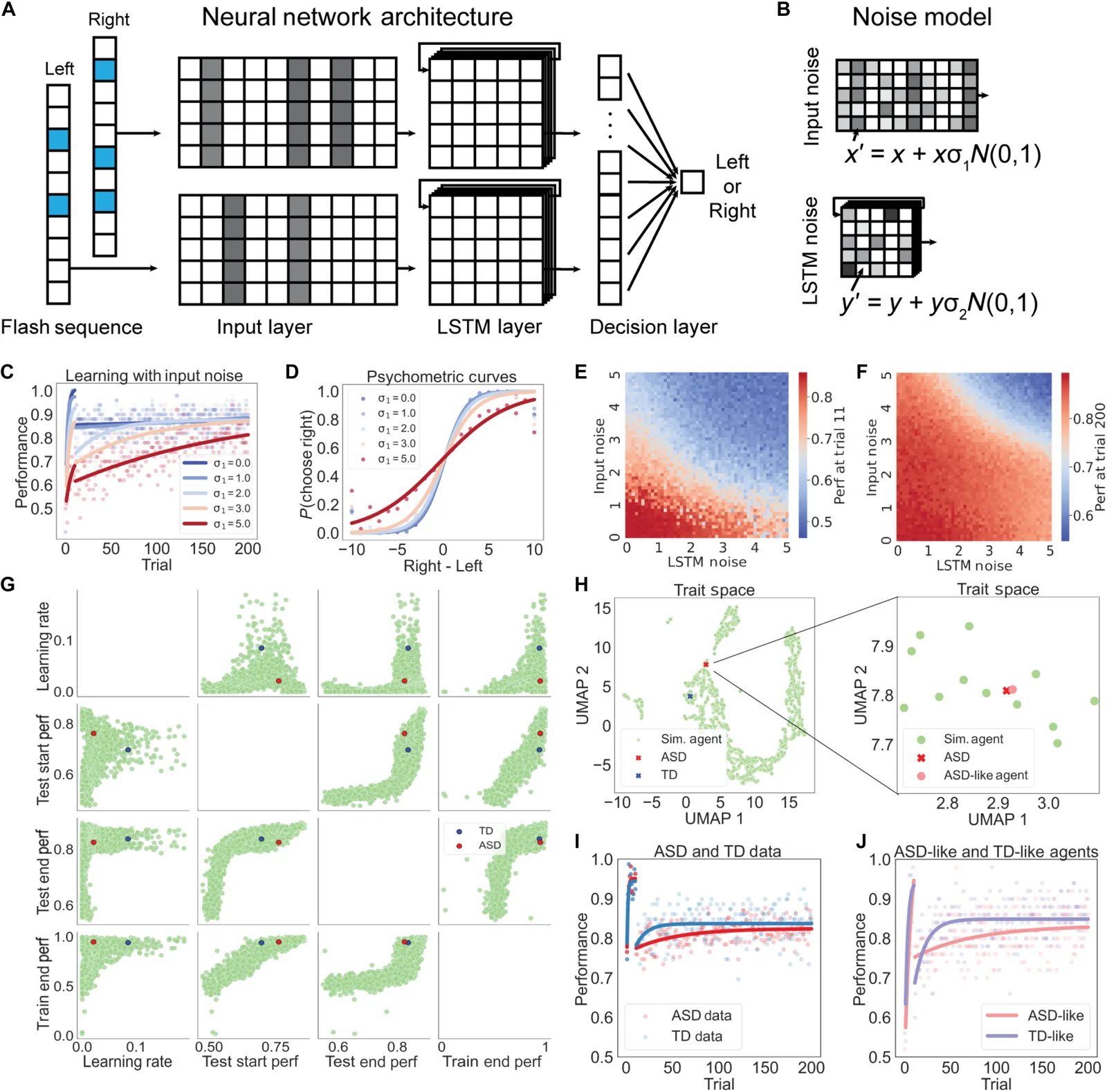
Using artificial learning agents to model ASD traits. (**A**) Neural network architecture for artificial learning agents. (**B**) Gaussian noise was injected into the input layer and LSTM layer. (**C**) Learning curve with varying input noise levels (σ_1_ = 0, 1, 2, 3, and 5) across 200 trials, showing that artificial agents could be trained to perform the task using the same curriculum as human participants, and input noise altered both the rate of learning and plateau performance. (**D**) Psychometric curves fitted to models with different noise levels. (**E** and **F**) Heatmaps showing accuracy at trial 11 (E) and trial 200 (F) for agents with varying levels of input and LSTM noise. (**G**) Scatterplot illustrating the relationship between different performance metrics (traits) of simulated agents (green dots), with average traits for ASD (red) and TD (blue) participants. (**H**) Left: UMAP of simulated agents in trait space, with ASD (red) and TD (blue) plotted within this space. Right: Zoomed-in visualization of the ASD-like agent, identified via Euclidean distance search in trait space. (**I**) Performance over 200 trials for ASD (red) and TD (blue), with fitted learning curves. (**J**) Performance over 200 trials for ASD-like and TD-like agents, identified from the closest simulated matches, with fitted learning curves for comparison.

To better understand the relationship between perceptual deficits and slower learning in our task, we modeled the behavior of TD and ASD populations using artificial neural network-based agents (Fig. 6). Like the human participants, the goal of these agents was to learn to associate accumulated sensory cues with the correct binary choice using feedback. Unlike the SDT and hierarchical Bayesian models, which characterize perceptual sensitivity, the neural network model explicitly captures how task rules are acquired over trials. This allowed us to assess how noise in the integration process might relate to learning.

In these networks, the flash sequences for the left and right sides are processed through an input layer, followed by a long short-term memory (LSTM) layer and a fully connected decision layer that determines the choice (Fig. 6A). All layers are fully connected, allowing the model to integrate temporal and spatial information within and across trials. Initially, the parameters (weights between layers) are randomly assigned, and the agents must learn to perform the task using the same curriculum as the humans—10 training trials (level 1) followed by 190 test trials (levels 2 to 10). Learning occurs through feedback: At the end of each trial, the agent generates a decision, receives a reward signal (0 or 1), and updates its parameters using backpropagation with gradient descent.

To assess how noise affected the neural network’s performance, we injected Gaussian noise into early stages of processing: Input noise was added to the input layer, affecting sensory representations, whereas LSTM noise was injected into the recurrent layer, influencing temporal integration (Fig. 6B). Noise strength was controlled by the SDs, σ_1_ and σ_2_, respectively.

In the absence of noise (σ_1_ = σ_2_ = 0), the model rapidly learned the task, reaching 85.53% accuracy at trial 11 (Fig. 6C). However, when noise was introduced, the learning rate and performance of the model changed (Fig. 6, C and D). These effects followed a gradient, with increasing noise systematically altering both learning and integration (Fig. 6, E and F).

We simulated a set of artificial agents with varying noise levels and mapped their positions in trait space, a 5D space defined by behavioral performance on the task (i.e., accuracy at trials 10, 11, and 200 and learning rate; Fig. 6G). Next, we identified the agents most similar to ASD and TD humans using a nearest-neighbor approach (Fig. 6H). These ASD-like and TD-like agents captured several features of their human counterparts, including slower learning and lower peak performance in ASD participants (Fig. 6, I and J). Together, these results show that noise in the integration process is sufficient to reproduce both the slower learning and reduced asymptotic performance observed in ASD participants. By explicitly modeling learning over trials, the neural network framework extends the SDT account of perceptual deficits to explain how altered integration may shape learning dynamics similar to those observed in ASD.

## DISCUSSION

Our results reveal that deficits in perceptual integration in the visual domain are widespread across the ASD population. Model-based analysis suggested that these deficits stem from increased noise in the integration process, which grows nonlinearly with the magnitude of visual evidence. Furthermore, we demonstrate that introducing noise into the sensory representations of artificial neural networks induces ASD-like traits in these agents.

These advances were enabled by GEODE, an online game inspired by animal tasks and designed for inclusivity, scalability, and cross-species compatibility. GEODE allowed us to recruit a large, heterogeneous cohort and demonstrate that even individuals with profound autism could learn and play, producing psychophysical datasets linked to symptom severity.

Our results are consistent with a wide range of previous observations suggesting altered perception in ASD populations (*3*, *5*). Specifically, these findings are well aligned with a previous work arguing for higher internal noise (*37*) and poorer external noise filtering in ASD (*38*), which reduces the fidelity of the evidence stream. However, we note that the increase in integration noise we report should be interpreted as a computational-level mechanism (*39*, *40*) rather than a claim about a single underlying psychological or neural circuit-level mechanism. In principle, noisier integration could arise from several factors, including degraded sensory representations of the flashes, fluctuations in sustained attention or limitations in short-term memory for accumulated evidence. The present model design does not uniquely isolate these processes, and does not provide decisive evidence for a specific circuit-level deficit. Nonetheless, the results do constrain the space of explanations and provide a foundation for further investigation. Future work combining GEODE with tasks that differentially tax sensory encoding, attentional control, and working memory, as well as circuit-level studies in animal models performing the same task, will be essential to pinpoint which neural systems give rise to the elevated integration noise we observe.

Although the precise circuit origin of increased integration noise remains unresolved, prior studies implicate variability in cortical sensory responses (*12*, *41*, *42*). One proposed source of this variability is an altered excitatory-to-inhibitory ratio, which can drive unstable neural dynamics as excitation, or gain, increases (*43*, *44*). Computational models suggest that gain fluctuations can generate the multiplicative noise observed here (*45*) and support a framework in which ASD risk genes alter neural gain, increasing cortical response variability and leading to altered perception (*13*, *37*, *44*).

Our results also reveal deficits in how nonsensory information is combined with sensory integration to drive decisions. Modeling revealed differences across ASD in bias-related parameters (e.g., side bias and win-stay), which reflect systematic choice strategies and sensitivity to recent outcomes, distinct from the fidelity of sensory evidence. These findings indicate that, in addition to increased integration noise, decision-making in ASD may be influenced by altered weighting of prior choices and feedback. This pattern could be viewed as broadly consistent with theories of altered use of priors in ASD (*46*–*50*). However, caution in this interpretation is warranted as our study was not designed to probe these effects.

It is important to note that perception in ASD is highly heterogeneous, varying along both variability across individuals and how individuals respond to task demands. For example, studies examining visual search have reported that ASD individuals outperform TD peers (*51*–*53*). One proposed explanation for this advantage is atypical sensitivity to local visual details. Consistent with this possibility, recent studies in rodent models of ASD have demonstrated altered visual feature sensitivity (*54*, *55*).

A remaining question is whether the deficits observed here are specific to ASD or reflect a dimension of impairment which could cut across NDDs. ASD frequently co-occurs with intellectual disability and other conditions that affect attention and working memory. Notably, in our sample, the strongest survey correlate of GEODE performance is adaptive functioning as measured by the VABS-3, rather than autistic symptom severity per se. Furthermore, our VABS-3 matching analysis (fig. S9) revealed that when ASD and TD participants were equated on adaptive functioning, differences in game-derived metrics were reduced. Thus, the degree of integration noise may track overall functional impairment more closely than the diagnostic category alone. However, these survey and matching results should be interpreted cautiously because VABS-3 matching necessarily reduces statistical power and because adaptive functioning overlaps with core and associated features of ASD, making it difficult to fully separate diagnostic status from broader functional impairment. Regardless of how these surveys are interpreted, GEODE robustly captures a perceptual-cognitive deficit in ASD that may also be present in other NDDs. We view this as a strength of GEODE: By providing a scalable, game-based assay that can be implemented in minimally verbal and cognitively impaired participants, it offers a framework for comparing computational mechanisms across ASD and other NDDs and for linking human behavior to circuit-level analyses in animal models using the same task.

In this study, we focused on adolescents due to their high representation in patient registries and their proficiency with digital interfaces. This age group represents a critical developmental window where objective phenotyping could help assess the effect of interventions and predict support needs during the transition to independence (*56*). An intriguing question is whether GEODE, or aspects of GEODE, could be extended to younger populations. A recent work suggests that children as young as 18 months can successfully engage with tablet-based assays (*57*). We speculate that future iterations of GEODE could be optimized for populations younger than demonstrated here. Optimizations could include shortening sessions through improved game flow and simplified motor mechanics using larger targets.

Beyond autism, this work also illustrates a broader methodological framework: adapting animal-derived tasks into accessible, scalable online paradigms. This approach provides a valuable tool for studying cognition and perception in other neurodevelopmental and psychiatric disorders, such as schizophrenia (*58*). Aligning research across humans and animal models will facilitate the discovery of neural circuit mechanisms that link genetic alterations to behavioral phenotypes, and these discoveries may ultimately guide the development of more effective interventions (*30*, *31*, *59*).

## MATERIALS AND METHODS

### Participants

All procedures were approved by the institutional review board at Boston University (IRB #6236E). We recruited *N* = 218 adolescents (11 to 17 years, inclusive) with an ASD diagnosis and *N* = 99 TD adolescent siblings. ASD and TD participants were recruited from the same four databases: (i) SPARK Research Match (Simons Powering Autism Research for Knowledge; *N* = 166), (ii) CARE at Boston University (*N* = 46), (iii) Simons Searchlight (*N* = 3), and (iv) the PMSF data hub (*N* = 3). ASD diagnoses was confirmed by SPARK, Searchlight, and PMSF. Alternatively, a community diagnosis was confirmed by the parent/caregiver. An initial screening was conducted with the parent/caregiver to determine whether the participant may be able to play the game with minimal support. Informed consent/assent were obtained from both the participant and the parent/caregiver. The parent/caregiver also provided demographic information about the participant and completed four surveys: (i) SRS-2, which was used to further support ASD diagnosis and provided a quantitative measure for ASD symptom severity; (ii) VABS-3, which measured overall adaptive skills and was used as a proxy for IQ; (iii) AASP, which measured sensory symptoms; and (iv) BIS, which measured inflexibility or rigidness of behavior. All participants were required to have normal or corrected-to-normal vision. Participants with a seizure disorder were excluded from the study.

*N* = 6 ASD and *N* = 3 TD participants were excluded from further analysis due to either data error or a nontypical SRS-2 score; for ASD, SRS-2 < 20 was considered nontypical; and for TD, SRS-2 > 120 was considered nontypical. Task success was determined by the following: completion of all 200 trials, and average accuracy of 61.6% or above for trials 11 to 200, this was based on the upper limit of the binomial 99.9% CI for chance performance. *N* = 89 TD and *N* = 174 ASD participants achieved this criterion. Furthermore, to separately evaluate the performance of more or less able ASD participants, we used their VABS-3 composite standard scores; ASD participants with a score of 75 or above were in the more able group, those below 75 were considered less able with low-adaptive skills, including some with profound ASD. Note that *N* = 17 ASD participants had missing VABS-3 scores, *N* = 11 TD participants also had either missing or unusually (VABS-3 < 75) low scores. These participants were excluded from analyses in which VABS-3 scores were used. To test whether participants who did not meet task criteria could do so on a second attempt, we retested a subset of ASD individuals (*N* = 11 individuals; see fig. S1). To gauge the reliability of the game-based measures across multiple game plays, we retested a subset of ASD and TD individuals who did achieve criterion on the first attempt (*N* = 35 individuals; 23 ASD and 12 TD; see fig. S7).

### Video game development

The online video game was inspired by a pulse-based evidence accumulation task for rodents (*60*–*62*) and modified from an earlier version used in humans (*32*). The game, playable on a computer as well as a tablet, has many visual details and a background narrative. It is a quest to collect gems. The task underlying the game includes streams of visual flashes. The flashes appear in 250-ms time bins. For a single trial, there could be up to 10 flashes on each side. Each trial was divided into three distinct states—trials began in the initial state, when the participant clicks or touches on the center of the screen. The trial then enters the cue state in which the participant is presented with the flash stimuli (in the shape of the gems). The cue state lasts for 2.5 to 3 s, after which the trial enters the decision state, during which the participant makes a left or right decision.

Postdecision, participants are provided with feedback in terms of point bonuses for correct choices or no points for erroneous choices. For correct responses, shiny gems magically appear and fly into the player’s treasure bag on the screen and the player’s point value increases. For incorrect choices, the gem remains hidden inside the rock formation and slowly disappears from view, resulting in a longer delay before the next trial.

We did not directly measure participants’ subjective interpretation of the reward or the point system. Instead, we relied on behavioral indicators of the effect of reward, including trial completion and accuracy. A previous work using a similar paradigm demonstrated that deterministic reward-like feedback supports learning of the decision rule (*32*). Participants trained with experiential feedback alone rapidly learned the task and displayed strategies similar to those who received explicit rule descriptions, whereas a cohort receiving random rewards, in which feedback was uncoupled to the choices, showed markedly reduced accuracy. These results highlight the importance of feedback-driven reward signals for learning in this paradigm.

Game progress is conveyed with a graphical treasure map, presented from time to time. The game had a total of 200 trials, and the first 10 trials were used for training: There were 10 versus 0 flashes or 9 versus 1 flashes on training trials. After training, participants were presented with the full stimulus set, where, on average, the flash ratio was 70:30. Flashes were generated using a Poisson process, and there could be a flash on either side, or both, or none, for a particular time bin.

### Data collection

Data were collected online using a split screen video call (Zoom). At the beginning of a session, the experimenter confirmed the participants’ information and then forwarded the link to play the game. Participants were provided with a deidentified subject ID with which they used to log into the game. Parents/caregivers received the following script:

“First, we will go through the informed consent form (ICF). I will walk you through this and please feel free to ask any questions as they come up. If you would still like to participate once we’ve gone through the ICF, you can go ahead and sign whenever you are ready. Although your teenager is playing the video game, we are going to have you fill out some questionnaires about your teenager’s behaviors. This should take around 30 min as they are all fairly short. If you have any questions about the various forms, feel free to ask me at any point. Once you finish the questionnaires, you are all set.”

Participants were not provided with instructions on how to play the game but were prompted on how to start the game using the following script:

“GEODE is a study where you will be playing a video game. I am going to explain the game a bit to you now! I am not going to tell you very much about it, but I will show you how it works. Then, I want you to see whether you can figure out how to win the game! You have about 45 min to play.”

Or, for adolescents with lower verbal abilities:

“GEODE is a study where you will be playing a video game. I am going to explain the game a bit to you now! When you click the screen, some flashes will come up and from there you have to decide what to do!”

On the first few training trials, the experimenter provided verbal encouragement such as “Good job” for a correct response or “Oops” for an incorrect response. The experimenter allowed the parent/caregiver to be present during game play to provide additional support. Occasionally, the experimenter gave out the rule of the game (go with the side with more flashes) upon participant request. There were also a few instances where the parent/caregiver provided some guidance. Nonetheless, all these instances were logged in detail and accounted for post hoc in the analysis of the data. After completing the game, participants were thanked for their participation and rewarded with an Amazon gift card worth $25. If both siblings were invited to participate, sessions were held at different times/days.

The demographic information and survey responses were collected using REDCap (Research Electronic Data Capture) a secure online data management system (*63*). Responses made in the game, along with the game variables, were collected using the MongoDB platform (https://mongodb.com/). Video and audio of the zoom sessions were also recorded and stored in a secured database. All data were deidentified.

### Data analysis

Data analyses were conducted using R Version 4.2.1; all figures were generated using R package ggplot2 (*64*). All deidentified data and code needed to create the figures in this paper can be found here: doi.org/10.5281/zenodo.16998131.

#### Analysis of survey scores

We followed published guidelines for calculating the scores for the four different surveys conducted in this study. SRS-2 is a 65-item questionnaire, each question is answered on a Likert scale, individual item scores are within the range of 0 to 3, some items (3, 7, 11, 12, 15, 17, 21, 22, 26, 32, 38, 40, 43, 45, 48, 52, and 55) were reverse scored, and missing item scores were replaced with the median values; items 52 and 55 had a median value of 2, whereas items 3, 5, 7, 9, 11, 12, 15, 17, 19, 21, 22, 25, 26, 28, 31, 38, 40, 43, 45, 48, and 56 has a median value of 1; all other items has a median value of 0. After these adjustments, the total score was calculated. SRS-2 total score served as an index for confirming ASD diagnosis, and thus, TD participants with extremely high SRS-2 scores (above 120) or ASD participants with very low scores (below 20) were considered as outliers. VABS-3 is a 120-item questionnaire, items are divided into three domains: communication, socialization and daily living skills. After calculating the raw totals for each domain, these were converted into standardized scores, which corrects for age. Then, the standardized scores from three domains were used to calculate the Adaptive Behavior Composite (ABC) score, which was used in the analyses. AASP is a 60-item questionnaire, divided into four quadrants: low registration, sensory seeking, sensory avoiding, and sensory sensitivity. We used the raw totals for each quadrant. Last, BIS is a 38-item questionnaire, the raw total score was used for BIS. Participants with missing SRS-2 scores were excluded from all analyses. Participants with missing VABS-3, AASP, or BIS scores were removed only from analyses that included those measures.

#### Division of participant groups

Our main groups of interest were ASD and TD. As mentioned above, SRS-2 scores were used to support ASD diagnosis; a small number of participants with anomalous SRS-2 scores were excluded. We collected a heterogeneous sample of ASD participants, including participants with intellectual disability and who were minimally verbal. Thus, in analyses pertaining to group level differences, we divided the ASD group into two subgroups, based on their VABS-3 ABC scores, above or below 75 to capture more and less able participants. In our analyses pertaining to individual differences, these subgroups were dropped. Another division of participants was based on task success, participants who completed all 200 trials and achieved an average accuracy of 61.6% or above were successful at the task, and these participants were included in further analyses; average accuracy was the proportion of correct choices for trials 11 to 200.

#### Average accuracy during training and posttraining

Motivated from staircase training curricula used in rodent experiments with the same task, here we included 10 training trials at the beginning, the first five trials had 10 versus 0 flash, and the next five had 9 versus 1 flashes. All participants experienced the same flash numbers for each training trial. To analyze training data, we averaged trial-wise accuracy across participants in each group. An exponential learning curve of the form $P_{n}=P_{\infty }-\left(P_{\infty }-P_{0}\right)e^{-\alpha n}$ was fit to the average accuracy using nonlinear least square estimation, where *n* is the trial number, *P*_*n*_ is the accuracy on trial *n*, and *P*_∞_ and *P*_0_ are the asymptotic and initial accuracy, respectively.

For trials posttraining (trials 11 to 200), the flash numbers varied; although, on average, the flash probability on one side was *P* = 0.7, the flash probability on the other side was 1 − *P*. To look at accuracy posttraining, we binned trials for each participant. A rolling average of accuracy over binned trials was used, with a bin size of 50 trials.

#### Bootstrapping and learning curve analysis

We fit an exponential learning curve model to participants’ trial-by-trial accuracy during the hard phase of the task (posttraining phase) defined as *y* = *A*_0_ + (*P* − *A*_0_) * [1 − *e*^(−L*trial)^] where *y* is the accuracy at a given trial, *A*_0_ is the initial accuracy, *P* is the asymptotic (plateau) accuracy, and *L* is the learning rate. To estimate variability in these parameters at the group level, we performed bootstrap resampling by sampling participants with replacement within each group (ASD and TD), fitting the learning curve to each resampled dataset over 1000 iterations. Group differences in the resulting parameter distributions were assessed using the KS test.

#### Nonlinear mixed-effects learning curve models

Because KS tests compare distributions but do not model trial-level data, we additionally fit nonlinear mixed-effects models to estimate group-level differences in baseline performance and learning rate while accounting for repeated measures. We used an exponential model of the form: Accuracy(*t*) = 1 − (1 − *P*_0_) * exp(−α*t*), with group entered as a fixed effect on *P*_0_ and α and subject-level random effects on these parameters. Models were fit separately for trials 1 to 10 and trials 11 to 200 to distinguish early acquisition from late learning dynamics.

#### Trial 11 transition and early-learning predictors

To quantify the training-to-generalization transition, we computed the change in accuracy from trial 10 to trial 11 for each participant and summarized group means. To assess whether early-learning dynamics predicted trial 11 performance, we fit subject-level linear models of accuracy as a function of trial over trials 1 to 10, extracting early-learning slopes and early mean accuracy. These metrics were entered into a regression model predicting trial 11 accuracy of the form: Accuracy11 ∼ Early Mean + Early Slope + Group.

#### Early-learning heterogeneity

To quantify individual variability in early acquisition, we restricted data to trials 1 to 10 and fit a mixed-effects logistic regression of the form: score ∼ trial + (trial | subject), allowing each participant to have their own learning slope. We extracted subject-level random slopes as individual early-learning estimates and compared their distributions across groups using a nonparametric Kruskal-Wallis test (with post hoc pairwise tests where appropriate). To test whether the ASD slope distribution reflected multiple latent subgroups, we fit Gaussian mixture models to the ASD slopes (using mclust), selected the best-fitting number of components by BIC, and compared to a single-component model to assess evidence for multiple slope components.

#### Psychometric functions

We used psychometric functions to look at the relation between choice/accuracy and the sensory information, i.e., the flash numbers. Note that, on each trial, there could be, at most, 10 flashes per side. Trials with equal number of flashes on both sides were not rewarded. We fit a GLM to the psychometric data. The model estimated the probability of choosing the right side (π) as a weighted sum of side bias, flash difference (*R* − *L*), previous reward (*WS*), and previous omission (*LS*)

$$\text{log}\left(\frac{\pi }{1-\pi }\right)=\beta_{0}+\beta_{1}*\left(R-L\right)+\beta_{2}*\mathrm{WS}+\beta_{3}*\mathrm{LS}$$

Here, β_1_ estimates the slope of the psychometric. *WS* was coded as: *WS* = 1 for a previous rewarded right choice, *WS* = −1 for a previous rewarded left choice, and *WS* = 0 otherwise. Similarly, *LS* was coded as: *LS* = 1 for a previous unrewarded right choice, *LS* = −1 for a previous unrewarded left choice, and *LS* = 0 otherwise. The GLM was fit to pooled data from each participant group, and for each trial bin of consideration.

#### Flash weights on choice probability

To estimate the weight of each flash time bin on choice probability we used a binomial GLM, in which choice probability (π) was modeled as

$$\begin{array}{c} \text{log}\left(\frac{\pi }{1-\pi }\right)=\beta_{0}+\beta_{1}*\mathrm{bin}1_{R}+\beta_{2}*\mathrm{bin}2_{R}+\beta_{3}*\\ \mathrm{bin}3_{R}+\beta_{4}*\mathrm{bin}4_{R}+\beta_{5}*\mathrm{bin}5_{R}+\beta_{6}*\mathrm{bin}6_{R}+\beta_{7}*\\ \mathrm{bin}7_{R}+\beta_{8}*\mathrm{bin}8_{R}+\beta_{9}*\mathrm{bin}9_{R}+\beta_{10}*\mathrm{bin}10_{R}+\beta_{11}*\\ \mathrm{bin}11_{L}+\beta_{12}*\mathrm{bin}12_{L}+\beta_{13}*\mathrm{bin}13_{L}+\beta_{14}*\\ \mathrm{bin}14_{L}+\beta_{15}*\mathrm{bin}15_{L}+\beta_{16}*\mathrm{bin}16_{L}+\beta_{17}*\mathrm{bin}17_{L}+\\ \beta_{18}*\mathrm{bin}18_{L}+\beta_{19}*\mathrm{bin}19_{L}+\beta_{20}*\mathrm{bin}20_{L}+\beta_{21}*\mathrm{bin}21_{L} \end{array}$$

The bin parameters are binary, indicating whether there was a flash on a particular bin, each trial could be divided up into 10 time bins (250 ms each). The GLM was fit to pooled choice data from each participant group.

#### SDT model

We used a SDT framework to quantify how noise in sensory integration influences choice behavior (*32*). On each trial, participants observed flash sequences on the left and right sides and selected the side perceived to contain more flashes. Choice behavior was modeled as a noisy comparison of accumulated sensory evidence.

Let *R* and *L* denote the number of flashes presented on the right and left sides, respectively. The probability of choosing the right side is defined as the probability that the perceived difference between right and left evidence is greater than zero

P(choose right)=P[(R−L)>0]

We model this decision variable as Gaussian distributed, with mean equal to the difference in flash counts (*R* − *L*) and variance σ^2^, which we refer to as perceptual noise.

Perceptual noise was defined according to a scalar variability model

$$\sigma^{2}=k_{0}+k_{1}\left(R^{2}+L^{2}\right)$$

where *k*_1_ represents integration noise, controlling how perceptual variability scales with the magnitude of accumulated evidence, and *k*_0_ is an initial noise term capturing baseline variability when little or no sensory evidence is available.

The probability of choosing the right side on a given trial is therefore

$$P\left(\text{choose right}\right)=\phi \left[\frac{R-L}{\sqrt{k_{0}+k_{1}\left(R^{2}+L^{2}\right)}}\right]$$

where ϕ(.) denotes the cumulative distribution function of the standard normal distribution.

To account for systematic choice tendencies beyond perceptual noise, we included side bias and win-stay/lose-switch terms as additive components to the evidence *R* − *L*. Model parameters were fit to posttraining choice data using maximum likelihood estimation with limited-memory BFGS (L-BFGS-B) algorithm. During parameter estimation, *k*0 and *k*1 were restricted to the positive domain, whereas bias terms (bs, *b*1, and *b*2) were left unbounded. Analyses included all posttraining trials from participants who met task performance criteria and SRS-2 criteria.

#### Hierarchical Bayesian model

We used the RStan package to fit a hierarchical Bayesian model that embeds the SDT framework described above [see also (*32*)]. The SDT model defines, for each trial, the probability of choosing the right side as a function of the evidence difference (*R* − *L*) and the associated perceptual noise $\sigma^{2}=k_{0}+k_{1}\left(R^{2}+L^{2}\right)$.

In the hierarchical Bayesian model, each individual choice on trial *i* was modeled as a Bernoulli random variable with probability π_*i*_, where π_*i*_ is given by the SDT decision rule

$$\pi_{i}=\phi \left[\frac{R_{i}-L_{i}+b_{1,j}+b_{2,j}\mathrm{wi}n_{i-1}+b_{3,j}\mathrm{los}e_{i-1}}{\sqrt{k_{0}+k_{1,j}\left({R_{i}}^{2}+{L_{i}}^{2}\right)}}\right]$$

for subject *j*. Thus, the SDT model specifies the likelihood at the single-trial level, whereas the hierarchical structure governs how model parameters are distributed across subjects and groups.

Integration noise *k*_1_ was estimated at both the population and individual-subject levels, allowing for subject-specific variability around a group-level distribution. In contrast, the initial noise parameter *k*_0_ was estimated only at the population level. This choice was motivated by prior parameter-recovery analyses (*32*), which showed that *k*_1_ can be reliably recovered at the individual level, whereas *k*_0_ cannot, despite significantly improving overall model fit when included at the group level. Side bias *b*_1_, win-stay *b*_2_, and lose-switch *b*_3_ parameters were estimated at the individual level.

Priors for *b*_1_, *b*_2_, and *b*_3_ were standard normal distributions, whereas *k*_0_ and *k*_1_ were modeled as log-normal distributions with population-level means and SDs. The hierarchical Bayesian model was fit separately for ASD and TD groups using posttraining trials from participants who met task performance criteria. Group comparisons were assessed using permutation tests to correct for multiple comparisons.

### Neural network simulation

#### Neural network architecture and training

To model learning in a sequential decision-making task, we implemented an artificial neural network with a dual-stream architecture, consisting of an input embedding layer, two independent LSTM layers, and a fully connected decision layer. The network processes sequences of visual stimuli and learns to make binary decisions based on these inputs.

1) Input Representation. The model processes two sequences of flash stimuli, one corresponding to the left side and one to the right. These sequences are first mapped into a continuous representation using two independent embedding layers. (i) The input size is 2, representing two binary flash sequences. (ii) Each input is embedded in a 64D space. (iii) The two embedding layers do not share weights, ensuring separate feature extraction for the two input streams.

2) LSTM Layers for Temporal Processing. After embedding, each sequence is passed through an independent LSTM layer. (i) Each LSTM layer has a hidden state dimension of 64. (ii) The two LSTMs operate in parallel, processing the left and right input streams separately. (iii) These LSTMs enable the model to integrate information across time steps and extract temporal patterns.

3) Fully Connected Decision Layer. The final decision is computed using a fully connected (linear) layer. (i) The outputs from both LSTMs are concatenated into a single feature vector. (ii) Given that each LSTM processes 20 time steps (10 for each flash sequence), the concatenated input to this layer has a total size of 20 × 64 + 20 × 64 = 1280. (iii) The output dimension is 2, representing the probability of choosing the left or right option. (iv) A softmax activation function is applied to obtain final decision probabilities.

The model is trained over 200 trials, with learning occurring after each trial. The training follows these steps:

1) Evaluation Phase. (i) The model processes the input sequences and makes a binary decision (left or right). (ii) The decision is compared to the correct answer, and the model receives a score of 0 (incorrect) or 1 (correct).

2) Learning Phase (Backpropagation). (i) Immediately after evaluating a trial, the model performs backpropagation based on the outcome of that specific trial. (ii) The network updates its weights accordingly before proceeding to the next trial.

This trial-by-trial learning setup allows the model to adapt incrementally, reflecting a continuous learning process similar to what happened during the video game that humans played.

#### Noise injection

To examine the effects of stochasticity in the sensory representation on learning performance, Gaussian noise was introduced at multiple stages of the neural network. Specifically, noise was applied to both the input embedding layer and the LSTM layer to investigate how variability at different processing stages affects learning dynamics.

Noise was generated by sampling random values from a standard normal distribution *N*(0,1) where the mean is 0 and the SD is 1. The noise was then scaled by a parameter σ, transforming the noise distribution to *N*(0,σ^2^). This transformation ensures that the injected noise retains a mean of 0 while allowing precise control over its SD through the parameter σ. The scaled noise was added directly to the input tensor *x* before being processed by the network.

To prevent the noise scaling process from interfering with gradient computations during backpropagation, the scaling factor σ was applied using a detached version of the input tensor. This ensures that the noise injection does not influence the optimization process, isolating its effects from the network’s learning dynamics.

Both input noise and LSTM noise were systematically varied across different conditions. Noise levels (σ) ranged from 0 to 5 in increments of 0.1, generating a wide spectrum of variability conditions for analysis.

#### Two-phase learning curve fitting

To quantify the relationship between trial number and accuracy, learning performance was modeled using an exponential learning curve function: *y* = *A*_0_ + (*P* − *A*_0_) * [1 − *e*^(−L*trial)^] where *y* is the accuracy at a given trial, *A*_0_ represents the initial accuracy, *P* is the plateau accuracy value, and *L* is the learning rate, controlling the rate of improvement.

To account for differences in learning dynamics between early training and later generalization, the learning curve was fitted separately for two trial segments:

1) Trials 1 to 10 (Training Phase): These trials consisted of easier training conditions (9 versus 1 and 10 versus 0), facilitating initial learning. The parameter *A*_0_ was fixed at 0.5, assuming chance-level performance at trial 1. The learning curve parameters extracted from this phase quantified the model’s adaptation to structured training.

2) Trials 11 to 200 (Testing Phase): Beginning at trial 11, the model was presented with the full stimulus set, requiring generalization beyond the initial training phase. A second learning curve was fitted to these trials to capture differences in long-term learning trajectories.

By fitting separate learning curves to the training and generalization phases, we obtained five parameters (*A*_0__test, *P*_train, *P*_test, *L*_train, and *L*_test) describing the learning behavior of each agent. These parameters were used as trait measures to characterize individual learning profiles.

#### Simulation design

To systematically examine the effects of noise on learning, both input noise and LSTM noise were varied across a range of conditions. Noise levels were incremented from 0 to 5 in steps of 0.1. Fifty agents were simulated for each noise condition. Learning curves were fitted to the averaged performance of each agent group.

This procedure generated a large-scale dataset of simulated agents, allowing detailed analysis of the impact of noise on learning performance.

In addition to the simulated agents, the same learning curve model was fitted to human behavioral data from TD and ASD participants. This enabled direct comparison between human learning traits and those of the neural network agents.

#### Nearest-agent matching using cKDTree

To identify simulated agents that best resembled the learning behavior of ASD and TD participants, we applied a nearest-neighbor search using the cKDTree algorithm from scipy.spatial.

Matching procedure

1) Each simulated agent was represented in a 5D trait space, where the dimensions corresponded to the fitted learning parameters (*A*_0__test, *P*_train, *P*_test, *L*_train, and *L*_test).

2) The ASD and TD participants’ empirical data were projected into this trait space based on their fitted learning parameters.

3) Using Euclidean distance, the closest simulated agents to the ASD and TD data points were identified.

4) These nearest-matching agents were then analyzed to assess how well they captured ASD-like and TD-like learning dynamics.

This approach allowed us to systematically compare simulated and human learning behaviors, providing insights into how different noise levels influence learning trajectories.

#### Dimensionality reduction with UMAP

To visualize the high-dimensional trait space, we applied uniform manifold approximation and projection (UMAP), reducing the 5D learning trait space to a 2D representation.

UMAP procedure

1) The five learning parameters extracted from the model were treated as feature vectors for each agent.

2) UMAP was used to reduce these vectors to a lower-dimensional space while preserving their structural relationships.

3) The projected 2D UMAP space was used to visualize the organization of ASD-like and TD-like agents.

4) Nearest simulated agents were identified in this space by previously computing Euclidean distances between ASD, TD, and the generated agents in the original 5D space.

## References

[1] American Psychiatric Association, *Diagnostic and Statistical Manual of Mental Disorders* (American Psychiatric Publishing Inc., ed. 5, 2013).

[2] E. J. Marco, L. B. N. Hinkley, S. S. Hill, S. S. Nagarajan, Sensory processing in autism: A review of neurophysiologic findings. Pediatr. Res. 69, 48R–54R (2011). 10.1203/PDR.0b013e3182130c54 PMC3086654 21289533

[3] C. E. Robertson, S. Baron-Cohen, Sensory perception in autism. Nat. Rev. Neurosci. 18, 671–684 (2017). 28951611 10.1038/nrn.2017.112

[4] L. Balasco, G. Provenzano, Y. Bozzi, Sensory abnormalities in autism spectrum disorders: A focus on the tactile domain, from genetic mouse models to the clinic. Front. Psych. 10, 1016 (2019). 10.3389/fpsyt.2019.01016 PMC6997554 32047448

[5] B.-S. Hadad, A. Yashar, Sensory perception in autism: What can we learn? Annu. Rev. Vis. Sci. 8, 239–264 (2022). 35804481 10.1146/annurev-vision-093020-035217

[6] Y. Morimoto, A. Imamura, N. Yamamoto, S. Kanegae, H. Ozawa, R. Iwanaga, “Atypical sensory characteristics in autism spectrum disorders” in *Autism Spectrum Disorders* (Exon Publications, 2021), pp. 55–66. 34495621

[7] E. Pellicano, L. Gibson, M. Maybery, K. Durkin, D. R. Badcock, Abnormal global processing along the dorsal visual pathway in autism: A possible mechanism for weak visuospatial coherence? Neuropsychologia 43, 1044–1053 (2005). 15769490 10.1016/j.neuropsychologia.2004.10.003

[8] C. E. Robertson, A. Martin, C. I. Baker, S. Baron-Cohen, Atypical integration of motion signals in autism spectrum conditions. PLOS ONE 7, e48173 (2012). 23185249 10.1371/journal.pone.0048173 PMC3502435

[9] J. I. Gold, M. N. Shadlen, The neural basis of decision making. Annu. Rev. Neurosci. 30, 535–574 (2007). 17600525 10.1146/annurev.neuro.29.051605.113038

[10] D. Poeppel, The analysis of speech in different temporal integration windows: Cerebral lateralization as “asymmetric sampling in time”. Speech Commun. 41, 245–255 (2003).

[11] A. Pirrone, I. Johnson, T. Stafford, E. Milne, A diffusion model decomposition of orientation discrimination in children with Autism Spectrum Disorder (ASD). Eur. J. Dev. Psychol. 17, 213–230 (2020).

[12] I. Dinstein, D. J. Heeger, L. Lorenzi, N. J. Minshew, R. Malach, M. Behrmann, Unreliable evoked responses in autism. Neuron 75, 981–991 (2012). 22998867 10.1016/j.neuron.2012.07.026 PMC3457023

[13] S. M. Haigh, Variable sensory perception in autism. Eur. J. Neurosci. 47, 602–609 (2018). 28474794 10.1111/ejn.13601

[14] D. R. Simmons, A. E. Robertson, L. S. McKay, E. Toal, P. McAleer, F. E. Pollick, Vision in autism spectrum disorders. Vision Res. 49, 2705–2739 (2009). 19682485 10.1016/j.visres.2009.08.005

[15] L. L. Orefice, A. L. Zimmerman, A. M. Chirila, S. J. Sleboda, J. P. Head, D. D. Ginty, Peripheral mechanosensory neuron dysfunction underlies tactile and behavioral deficits in mouse models of ASDs. Cell 166, 299–313 (2016). 27293187 10.1016/j.cell.2016.05.033 PMC5567792

[16] L. L. Orefice, Outside-in: Rethinking the etiology of autism spectrum disorders. Science 366, 45–46 (2019). 31604296 10.1126/science.aaz3880

[17] L. L. Orefice, Peripheral somatosensory neuron dysfunction: Emerging roles in autism spectrum disorders. Neuroscience 445, 120–129 (2020). 32035119 10.1016/j.neuroscience.2020.01.039 PMC7415509

[18] H. Tager-Flusberg, C. Kasari, Minimally verbal school-aged children with autism spectrum disorder: The neglected end of the spectrum. Autism Res. 6, 468–478 (2013). 24124067 10.1002/aur.1329 PMC3869868

[19] H. Tager-Flusberg, D. Plesa Skwerer, R. M. Joseph, B. Brukilacchio, J. Decker, B. Eggleston, S. Meyer, A. Yoder, Conducting research with minimally verbal participants with autism spectrum disorder. Autism 21, 852–861 (2017). 27354431 10.1177/1362361316654605 PMC6988898

[20] A. Thurm, A. Halladay, D. Mandell, M. Maye, S. Ethridge, C. Farmer, Making research possible: Barriers and solutions for those with ASD and ID. J. Autism Dev. Disord. 52, 4646–4650 (2022). 34716842 10.1007/s10803-021-05320-1 PMC9054938

[21] C. Lord, T. Charman, A. Havdahl, P. Carbone, E. Anagnostou, B. Boyd, T. Carr, P. J. de Vries, C. Dissanayake, G. Divan, C. M. Freitag, M. M. Gotelli, C. Kasari, M. Knapp, P. Mundy, A. Plank, L. Scahill, C. Servili, P. Shattuck, E. Simonoff, A. T. Singer, V. Slonims, P. P. Wang, M. C. Ysrraelit, R. Jellett, A. Pickles, J. Cusack, P. Howlin, P. Szatmari, A. Holbrook, C. Toolan, J. B. McCauley, The Lancet Commission on the future of care and clinical research in autism. Lancet 399, 271–334 (2022). 34883054 10.1016/S0140-6736(21)01541-5

[22] L. E. Wachtel, J. Escher, A. Halladay, A. Lutz, G. M. Satriale, A. Westover, C. Lopez-Arvizu, Profound autism: An imperative diagnosis. Pediatr. Clin. North Am. 71, 301–313 (2024). 38423722 10.1016/j.pcl.2023.12.005

[23] J. L. Silverman, A. Thurm, S. B. Ethridge, M. M. Soller, S. P. Petkova, T. Abel, M. D. Bauman, E. S. Brodkin, H. Harony-Nicolas, M. Wöhr, A. Halladay, Reconsidering animal models used to study autism spectrum disorder: Current state and optimizing future. Genes Brain Behav. 21, e12803 (2022). 35285132 10.1111/gbb.12803 PMC9189007

[24] K. Koldewyn, D. Whitney, S. M. Rivera, The psychophysics of visual motion and global form processing in autism. Brain 133, 599–610 (2010). 19887505 10.1093/brain/awp272 PMC2858014

[25] E. Milne, J. Swettenham, P. Hansen, R. Campbell, H. Jeffries, K. Plaisted, High motion coherence thresholds in children with autism. J. Child Psychol. Psychiatry 43, 255–263 (2002). 11902604 10.1111/1469-7610.00018

[26] K. J. Price, M. Shiffrar, K. A. Kerns, Movement perception and movement production in Asperger’s Syndrome. Res. Autism Spectr. Disord. 6, 391–398 (2012).

[27] R. Van der Hallen, C. Manning, K. Evers, J. Wagemans, Global motion perception in autism spectrum disorder: A meta-analysis. J. Autism Dev. Disord. 49, 4901–4918 (2019). 31489542 10.1007/s10803-019-04194-8 PMC6841654

[28] B. W. Brunton, M. M. Botvinick, C. D. Brody, Rats and humans can optimally accumulate evidence for decision-making. Science 340, 95–98 (2013). 23559254 10.1126/science.1233912

[29] G. A. Kane, R. A. Senne, B. B. Scott, Rat movements reflect internal decision dynamics in an evidence accumulation task. J. Neurophysiol. 132, 1608–1620 (2024). 39382979 10.1152/jn.00181.2024 PMC11573272

[30] H. C. Barron, R. B. Mars, D. Dupret, J. P. Lerch, C. Sampaio-Baptista, Cross-species neuroscience: Closing the explanatory gap. Philos. Trans. R. Soc. London Ser. B Biol. Sci. 376, 20190633 (2021). 33190601 10.1098/rstb.2019.0633 PMC7116399

[31] K. Schmack, T. Ott, Á. Kepecs, Computational psychiatry across species to study the biology of hallucinations. JAMA Psychiatry 79, 75–76 (2022). 34817545 10.1001/jamapsychiatry.2021.3200 PMC10375515

[32] Q. Do, Y. Li, G. A. Kane, J. T. McGuire, B. B. Scott, Assessing evidence accumulation and rule learning in humans with an online game. J. Neurophysiol. 129, 131–143 (2023). 36475830 10.1152/jn.00124.2022

[33] J. N. Constantino, C. P. Gruber, *Social Responsiveness Scale, Second Edition (SRS-2) Manual* (Western Psychological Services, 2012).

[34] S. S. Sparrow, D. V. Cicchetti, C. A. Saulnier, *Vineland Adaptive Behavior Scales, Third Edition* (Pearson, 2016).

[35] C. Brown, W. Dunn, *Adult/Adolescent Sensory Profile: User’s Manual* (Psychological Corporation, 2002).

[36] L. Lecavalier, J. Bodfish, C. Harrop, A. Whitten, D. Jones, J. Pritchett, R. Faldowski, B. Boyd, Development of the Behavioral Inflexibility Scale for children with autism spectrum disorder and other developmental disabilities. Autism Res. 13, 489–499 (2020). 31904198 10.1002/aur.2257 PMC8293897

[37] I. Dinstein, D. J. Heeger, M. Behrmann, Neural variability: Friend or foe? Trends Cogn. Sci. 19, 322–328 (2015). 25979849 10.1016/j.tics.2015.04.005

[38] W. J. Park, K. B. Schauder, R. Zhang, L. Bennetto, D. Tadin, High internal noise and poor external noise filtering characterize perception in autism spectrum disorder. Sci. Rep. 7, 17584 (2017). 29242499 10.1038/s41598-017-17676-5 PMC5730555

[39] T. Poggio, The *Levels of Understanding* framework, revised. Perception 41, 1017–1023 (2012). 23409366 10.1068/p7299

[40] D. Marr, *Vision: A Computational Investigation into the Human Representation and Processing of Visual Information* (MIT Press, 2010).

[41] E. Milne, Increased intra-participant variability in children with autistic spectrum disorders: Evidence from single-trial analysis of evoked EEG. Front. Psychol. 2, 51 (2011). 21716921 10.3389/fpsyg.2011.00051 PMC3110871

[42] P. M. Weinger, V. Zemon, L. Soorya, J. Gordon, Low-contrast response deficits and increased neural noise in children with autism spectrum disorder. Neuropsychologia 63, 10–18 (2014). 25107679 10.1016/j.neuropsychologia.2014.07.031

[43] J. L. R. Rubenstein, M. M. Merzenich, Model of autism: Increased ratio of excitation/inhibition in key neural systems: Model of autism. Genes Brain Behav. 2, 255–267 (2003). 14606691 10.1034/j.1601-183x.2003.00037.x PMC6748642

[44] V. S. Sohal, J. L. R. Rubenstein, Excitation-inhibition balance as a framework for investigating mechanisms in neuropsychiatric disorders. Mol. Psychiatry 24, 1248–1257 (2019). 31089192 10.1038/s41380-019-0426-0 PMC6742424

[45] R. L. T. Goris, C. M. Ziemba, J. A. Movshon, E. P. Simoncelli, Slow gain fluctuations limit benefits of temporal integration in visual cortex. J. Vis. 18, 8 (2018). 10.1167/18.8.8 PMC6107324 30140890

[46] L. Fazioli, B.-S. Hadad, R. N. Denison, A. Yashar, Suboptimal but intact integration of Bayesian components during perceptual decision-making in autism. Mol. Autism. 16, 2 (2025). 39806435 10.1186/s13229-025-00639-3 PMC11731163

[47] J.-P. Noel, E. Balzani, L. Acerbi, J. Benson, International Brain Laboratory, C. Savin, D. E. Angelaki, A common computational and neural anomaly across mouse models of autism. Nat. Neurosci. 28, 1519–1532 (2025). 40461847 10.1038/s41593-025-01965-8 PMC12403184

[48] M. F. Davatolhagh, J. Couto, M. Melin, L. T. Oesch, C. Findling, P. Morales, I. Hong, International Brain Laboratory, A. K. Churchland, Altered use of prior expectations and modified neural dynamics in a mouse model of autism. bioRxiv 2025.09.12.675910 [Preprint] (2025). 10.1101/2025.09.12.675910.

[49] K. Cui, X. Lin, R. Gao, S. Jing, F. Luo, J. Wang, A systematic review and meta-analysis of empirical evidence for the simple Bayesian model of autism. Neuropsychol. Rev. 36, 184–195 (2026). 40576893 10.1007/s11065-025-09672-8

[50] N. Angeletos Chrysaitis, P. Seriès, 10 years of Bayesian theories of autism: A comprehensive review. Neurosci. Biobehav. Rev. 145, 105022 (2023). 36581168 10.1016/j.neubiorev.2022.105022

[51] M. A. O’Riordan, K. C. Plaisted, J. Driver, S. Baron-Cohen, Superior visual search in autism. J. Exp. Psychol. Hum. Percept. Perform. 27, 719–730 (2001). 11424657 10.1037//0096-1523.27.3.719

[52] R. M. Joseph, B. Keehn, C. Connolly, J. M. Wolfe, T. S. Horowitz, Why is visual search superior in autism spectrum disorder?: Visual search in ASD. Dev. Sci. 12, 1083–1096 (2009). 19840062 10.1111/j.1467-7687.2009.00855.x PMC12049234

[53] T. Gliga, R. Bedford, T. Charman, M. H. Johnson, BASIS Team, Enhanced visual search in infancy predicts emerging autism symptoms. Curr. Biol. 25, 1727–1730 (2015). 26073135 10.1016/j.cub.2015.05.011 PMC4502951

[54] N. Cheng, E. Pagtalunan, A. Abushaibah, J. Naidu, W. K. Stell, J. M. Rho, Y. Sauvé, Atypical visual processing in a mouse model of autism. Sci. Rep. 10, 12390 (2020). 32709898 10.1038/s41598-020-68589-9 PMC7381655

[55] R. T. Ash, G. Palagina, J. A. Fernandez-Leon, J. Park, R. Seilheimer, S. Lee, J. Sabharwal, F. Reyes, J. Wang, D. Lu, M. Sarfraz, M. Froudarakis, A. S. Tolias, S. M. Wu, S. M. Smirnakis, Increased reliability of visually-evoked activity in area V1 of the MECP2-duplication mouse model of autism. J. Neurosci. 42, 6469–6482 (2022). 35831173 10.1523/JNEUROSCI.0654-22.2022 PMC9398540

[56] K. Hume, B. A. Boyd, J. V. Hamm, S. Kucharczyk, Supporting independence in adolescents on the autism spectrum. Remedial Spec. Educ. 35, 102–113 (2014).

[57] S. Perochon, J. Matias Di Martino, K. L. H. Carpenter, S. Compton, N. Davis, S. Espinosa, L. Franz, A. D. Rieder, C. Sullivan, G. Sapiro, G. Dawson, A tablet-based game for the assessment of visual motor skills in autistic children. NPJ Digit. Med. 6, 17 (2023). 36737475 10.1038/s41746-023-00762-6 PMC9898502

[58] G. Horga, A. Abi-Dargham, An integrative framework for perceptual disturbances in psychosis. Nat. Rev. Neurosci. 20, 763–778 (2019). 31712782 10.1038/s41583-019-0234-1

[59] E. J. Nestler, S. E. Hyman, Animal models of neuropsychiatric disorders. Nat. Neurosci. 13, 1161–1169 (2010). 20877280 10.1038/nn.2647 PMC3750731

[60] B. B. Scott, C. M. Constantinople, J. C. Erlich, D. W. Tank, C. D. Brody, Sources of noise during accumulation of evidence in unrestrained and voluntarily head-restrained rats. Elife 4, e11308 (2015). 26673896 10.7554/eLife.11308 PMC4749559

[61] B. B. Scott, C. M. Constantinople, A. Akrami, T. D. Hanks, C. D. Brody, D. W. Tank, Fronto-parietal cortical circuits encode accumulated evidence with a diversity of timescales. Neuron 95, 385–398.e5 (2017). 28669543 10.1016/j.neuron.2017.06.013 PMC9453285

[62] B. B. Scott, S. Y. Thiberge, C. Guo, D. G. R. Tervo, C. D. Brody, A. Y. Karpova, D. W. Tank, Imaging cortical dynamics in GCaMP transgenic rats with a head-mounted widefield macroscope. Neuron 100, 1045–1058.e5 (2018). 30482694 10.1016/j.neuron.2018.09.050 PMC6283673

[63] P. A. Harris, R. Taylor, B. L. Minor, V. Elliott, M. Fernandez, L. O’Neal, L. McLeod, G. Delacqua, F. Delacqua, J. Kirby, S. N. Duda, REDCap Consortium, The REDCap consortium: Building an international community of software platform partners. J. Biomed. Inform. 95, 103208 (2019). 31078660 10.1016/j.jbi.2019.103208 PMC7254481

[64] H. Wickham, “Getting started with ggplot2” in *Use R!* (Springer International Publishing, 2016), pp. 11–31.

