# Supplementary material for: Computational mechanisms of perception in autism revealed using games inspired by rodent operant tasks
Source: Sci Adv. 2026 Sep 11;12(37):eaec9291. doi: 10.1126/sciadv.aec9291 (PMC13564830; doi:10.1126/sciadv.aec9291)
Supplement: Supplementary file 1 [file sciadv.aec9291_sm.pdf]

Science Advances
AAAS

Supplementary Materials for

Computational mechanisms of perception in autism revealed using games inspired by rodent operant tasks

Sucheta Chakravarty *et al.*

Corresponding author: Benjamin B. Scott, bbs@bu.edu

Sci. Adv. **12**, eaec9291 (2026)
DOI: 10.1126/sciadv.aec9291

This PDF file includes:

Figs. S1 to S9

Supplementary Figures

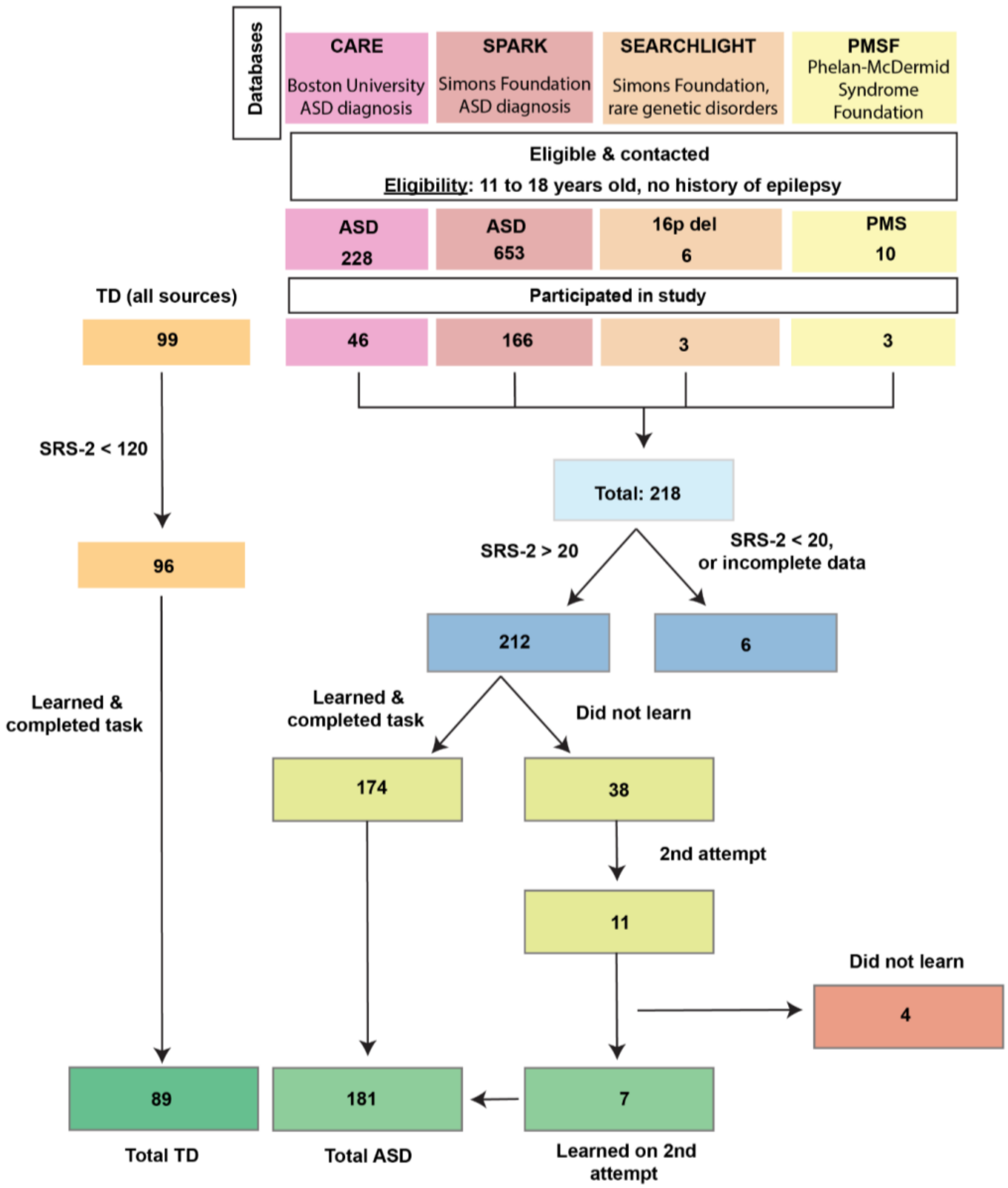

Figure S1. Recruitment. Participants were recruited from different databases, contacted through email advertisements. Eligibility was determined by age, ASD diagnosis and history of epilepsy. Those with missing game data were excluded. Participants with unusual SRS-2 scores, defined as SRS-2>120 for TD (N=3) and SRS-2<20 for ASD (N=6) were also excluded. Criteria for successful game play and learning were determined by 1) completion of all (N = 200) trials, and 2) average accuracy of 61.6% or above post training. A subset of ASD participants who failed to reach above criteria were invited back for another session (N=11).

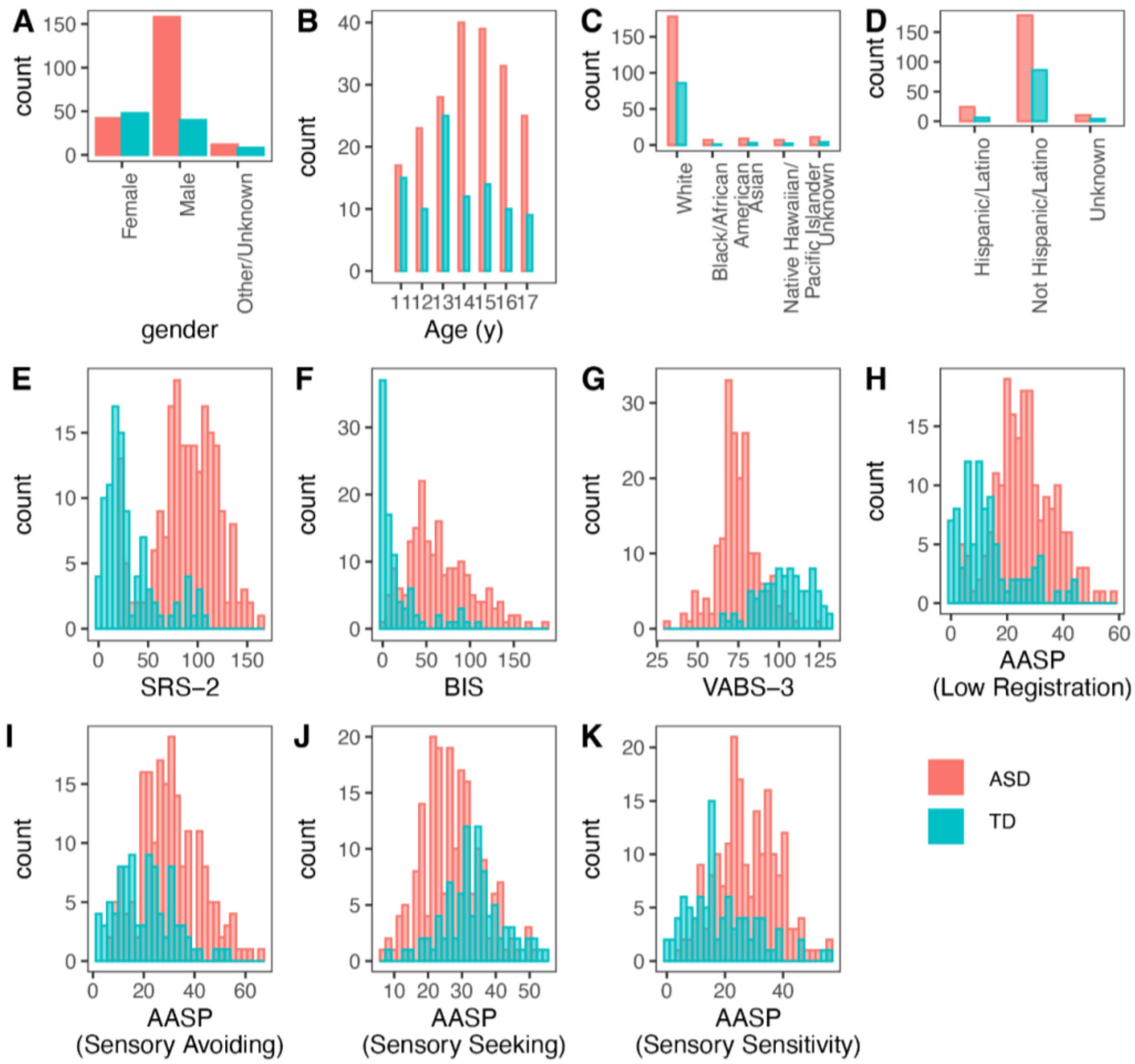

Figure S2. Participant demographics. Demographic distributions of our data indicate a large, heterogeneous sample of N=212 ASD and N=96 TD participants. **A.** ASD participants included a higher proportion of males, as commonly noted in relevant literature. **B.** We recruited ASD and TD participants across ages 11 to 17 years, though the ASD group was overall slightly older [p=0.01, mean age for ASD = 14 y, for TD = 13 y]. **C–D.** Distribution of race and ethnicity paralleled that of the US demographic. **E–G.** Distribution of SRS-2, BIS and VABS-3 scores suggested heterogeneity in symptom severity among ASD, also confirmed differences between TD and ASD. **H-K.** Distribution of scores from AASP, a questionnaire designed to assess characteristics of sensory experiences in ASD.

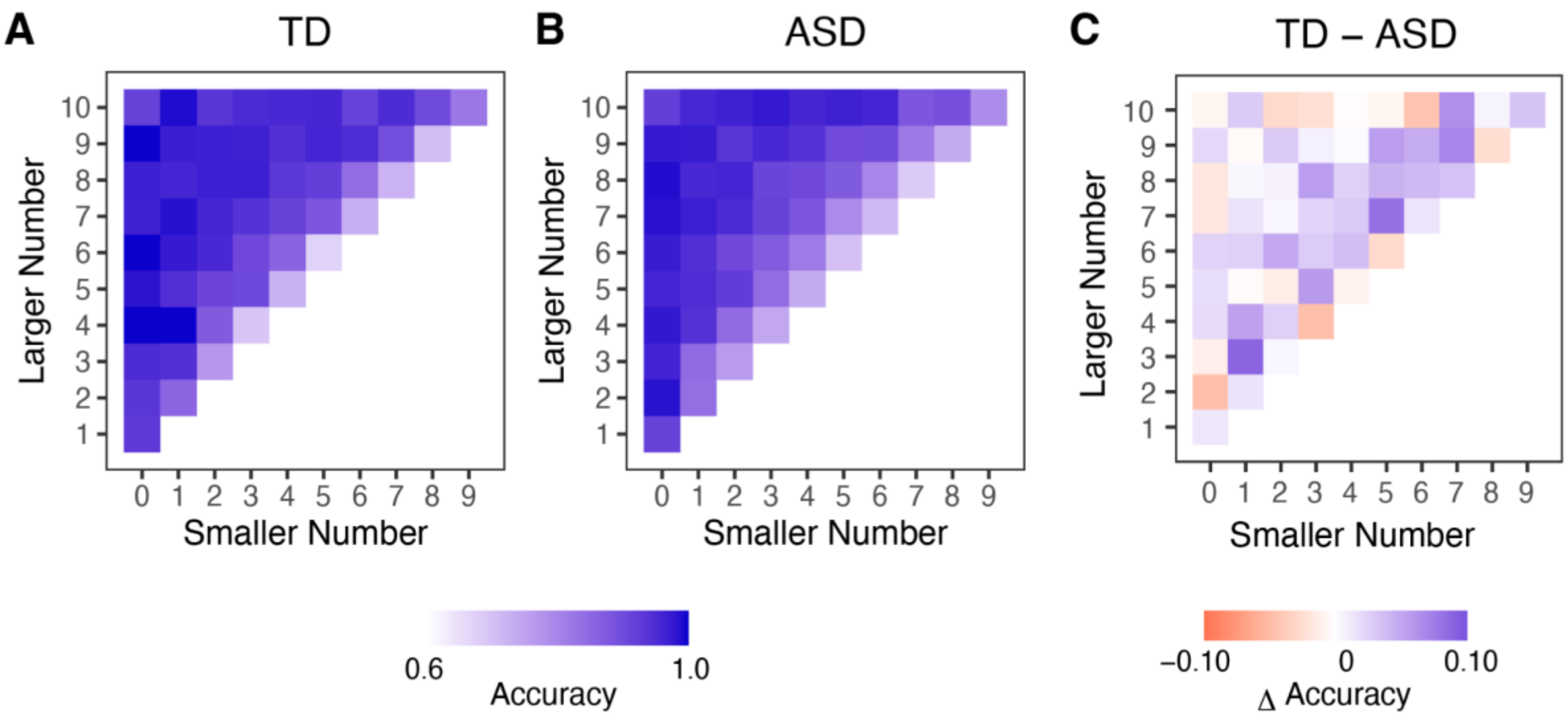

Figure S3. Psychometric performance. A–C. Two-dimensional psychometric function plot showing choice accuracy as a function of the number of flashes on each side, separately for TD **(A)**, all ASD **(B)**, and the difference between TD and ASD **(C)**.

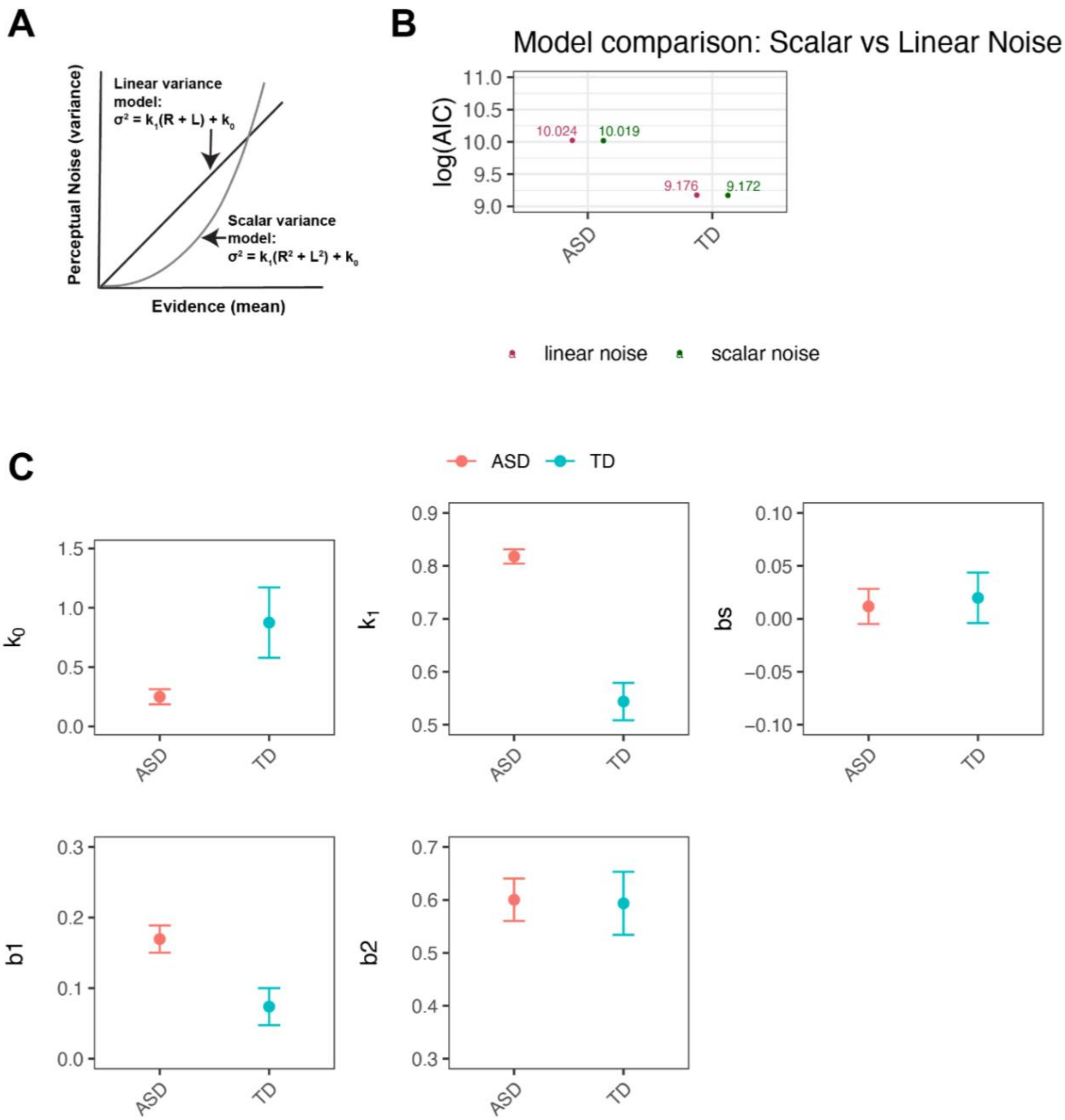

Figure S4. Linear vs Scalar variability signal detection theory models. A. Signal detection theory (SDT) models with linear and scalar noise. **B.** Comparison of the linear and scalar models for ASD and TD data. **C.** Model parameters for the linear noise model for ASD and TD.

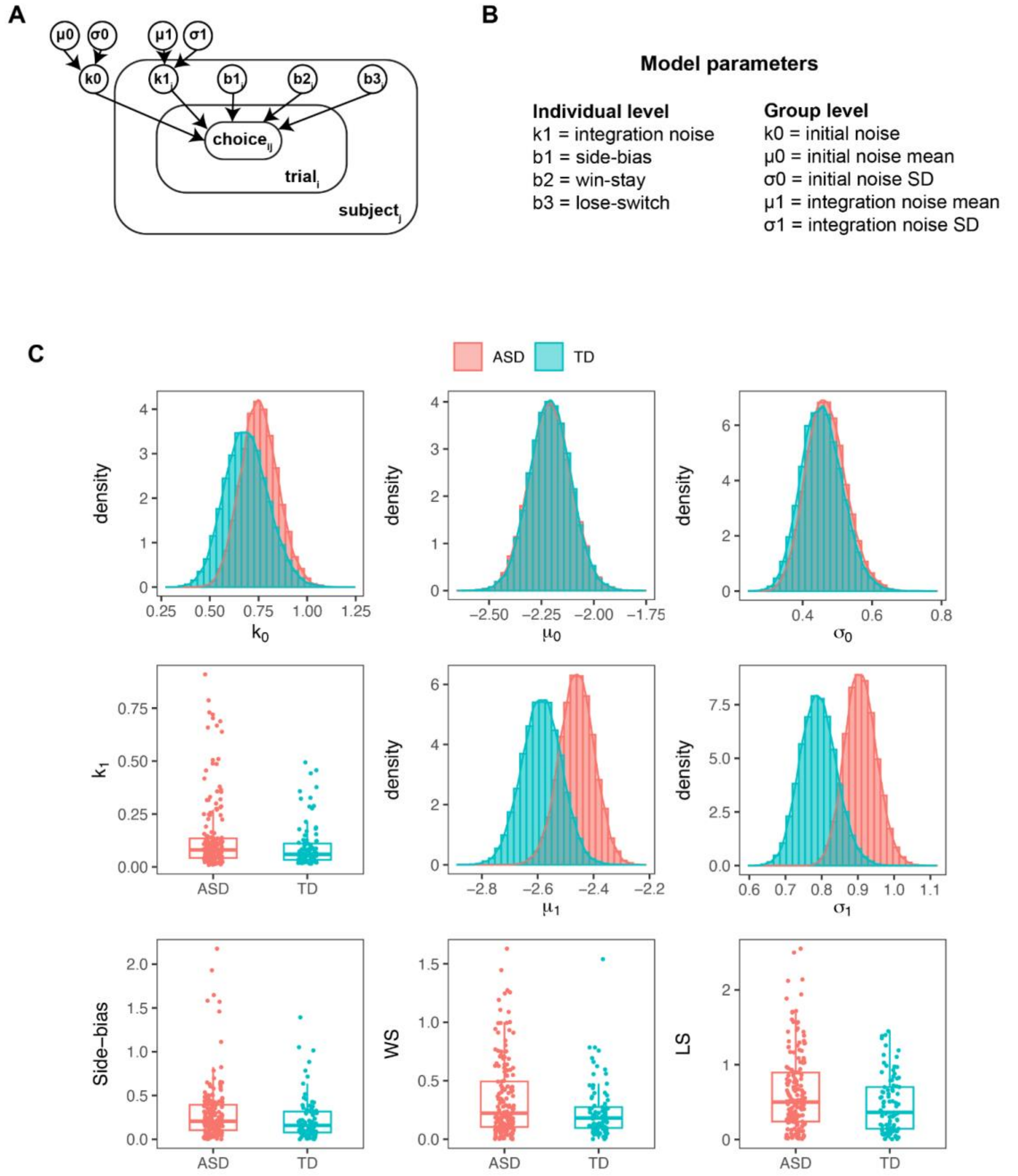

Figure S5. A hierarchical Bayesian model estimated individual differences in evidence accumulation. A. To fit the SDT model to individual participant data, we used a hierarchical Bayesian approach. **B.** The HBM-SDT model included different parameters, some estimated at the population level (histograms, indicating posterior probabilities), and some at the level of individual participants (box and whisker plots). The models were fit separately for ASD and TD. **C.** Fitted model parameter values suggested overall greater variability for ASD; significant group differences were also noted.

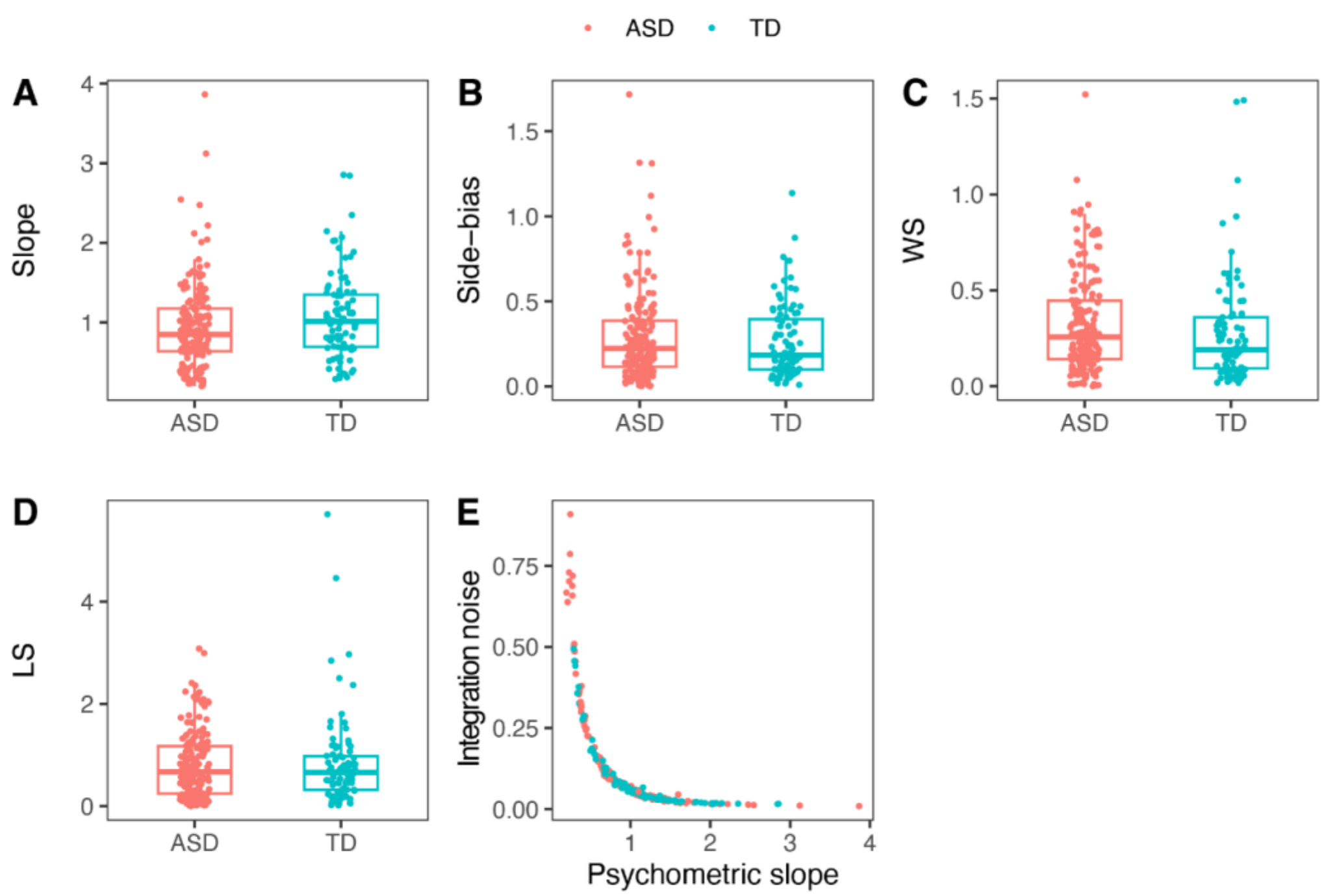

Figure S6. Individual participant psychometrics. A–D. We fit GLMs to individual participant data to estimate the psychometric slope, along with side bias, and trial history biases such as win-stay or lose-switch **E.** Correspondence between the psychometric slope estimated with GLM and integration noise ($k1$) estimated with HBM-SDT.

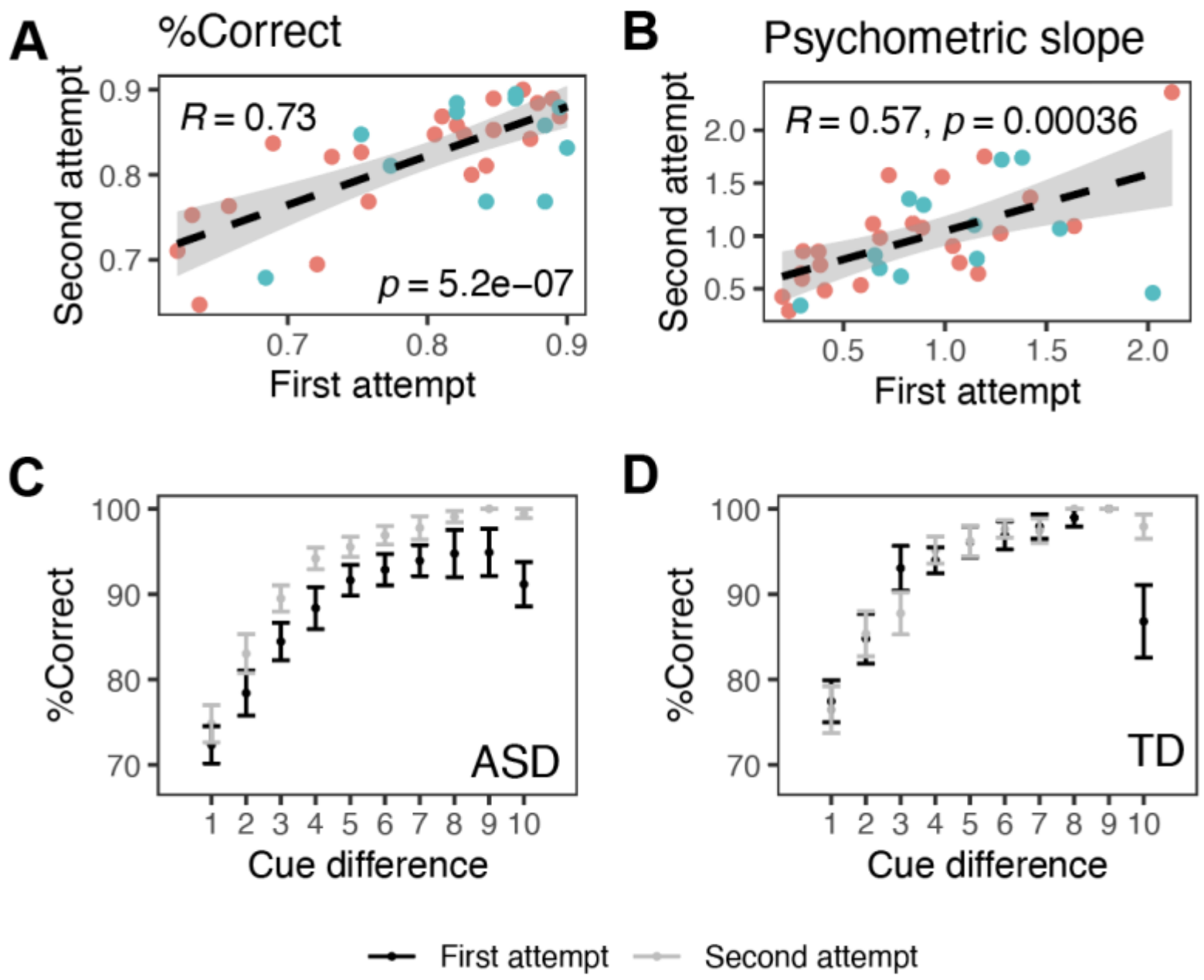

Figure S7. Retest reliability. 35 individuals (23 ASD; 12 TD) returned for a second session to estimate the retest reliability of the game. Accuracy (A) and the slope of the psychometric function (B) were highly correlated between the first and second attempt ($P<0.001$). Colored dots represent individual players, ASD in red, TD in blue. C) For the ASD cohort, accuracy in the second attempt was greater than the first. D) Psychometric performance across for the TD cohort was similar across first and second attempts.

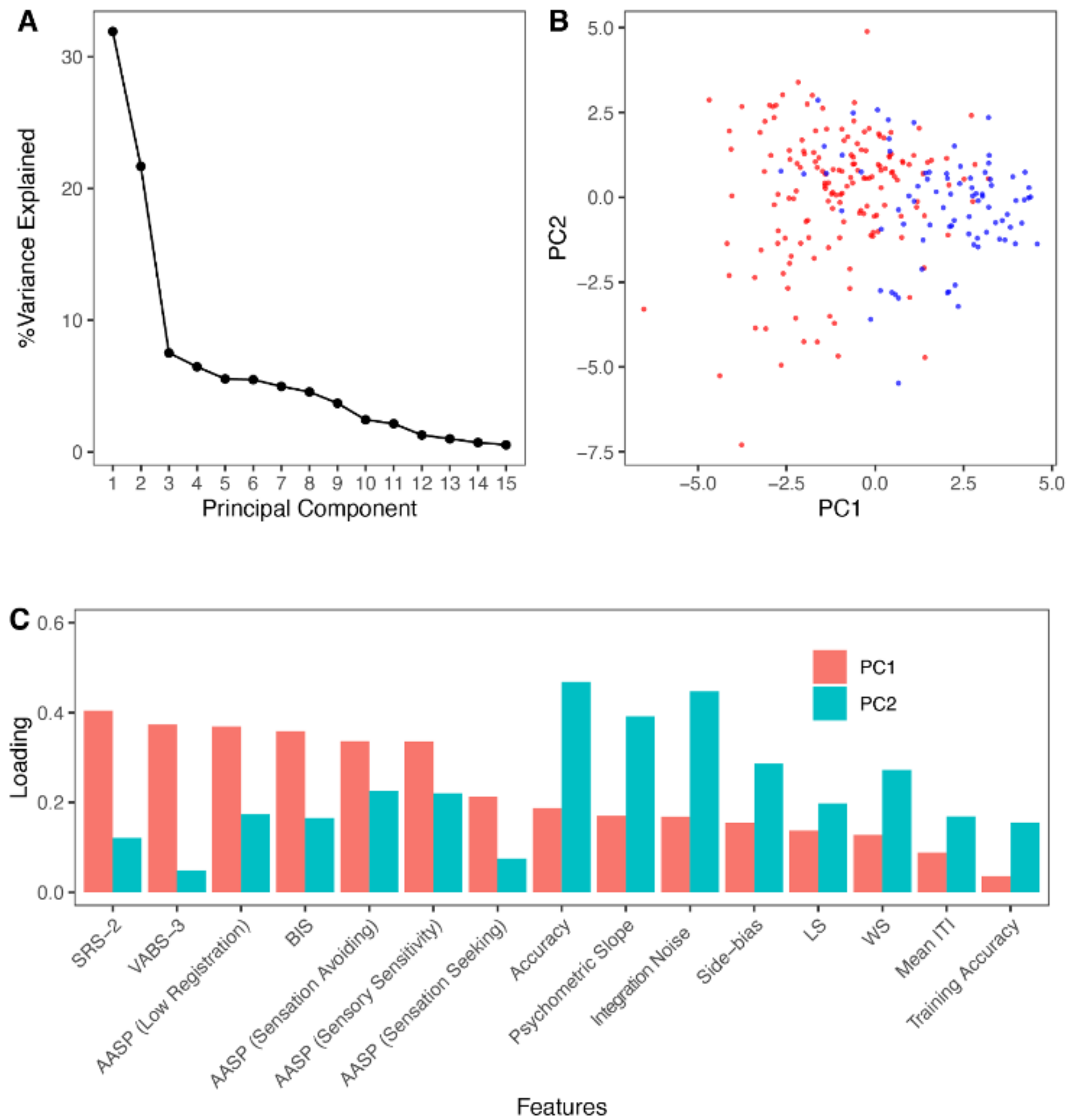

Figure S8. Game measures describe additional independent variance between ASD and TD. A. We conducted principal component analysis (PCA) on the combined (game and survey) features. Principal component (PC) 1 and PC2 explained most variance in the data. **B.** ASD individuals (red dots) and TD individuals (blue dots) were well separated in the space of the first two PCs. **C.** The survey measures loaded heavily onto PC1 (red) while the game measures loaded heavily onto PC2 (green). The PCA calculation did not use group information, this information was used to evaluate group differences in PC scores and for visualization.

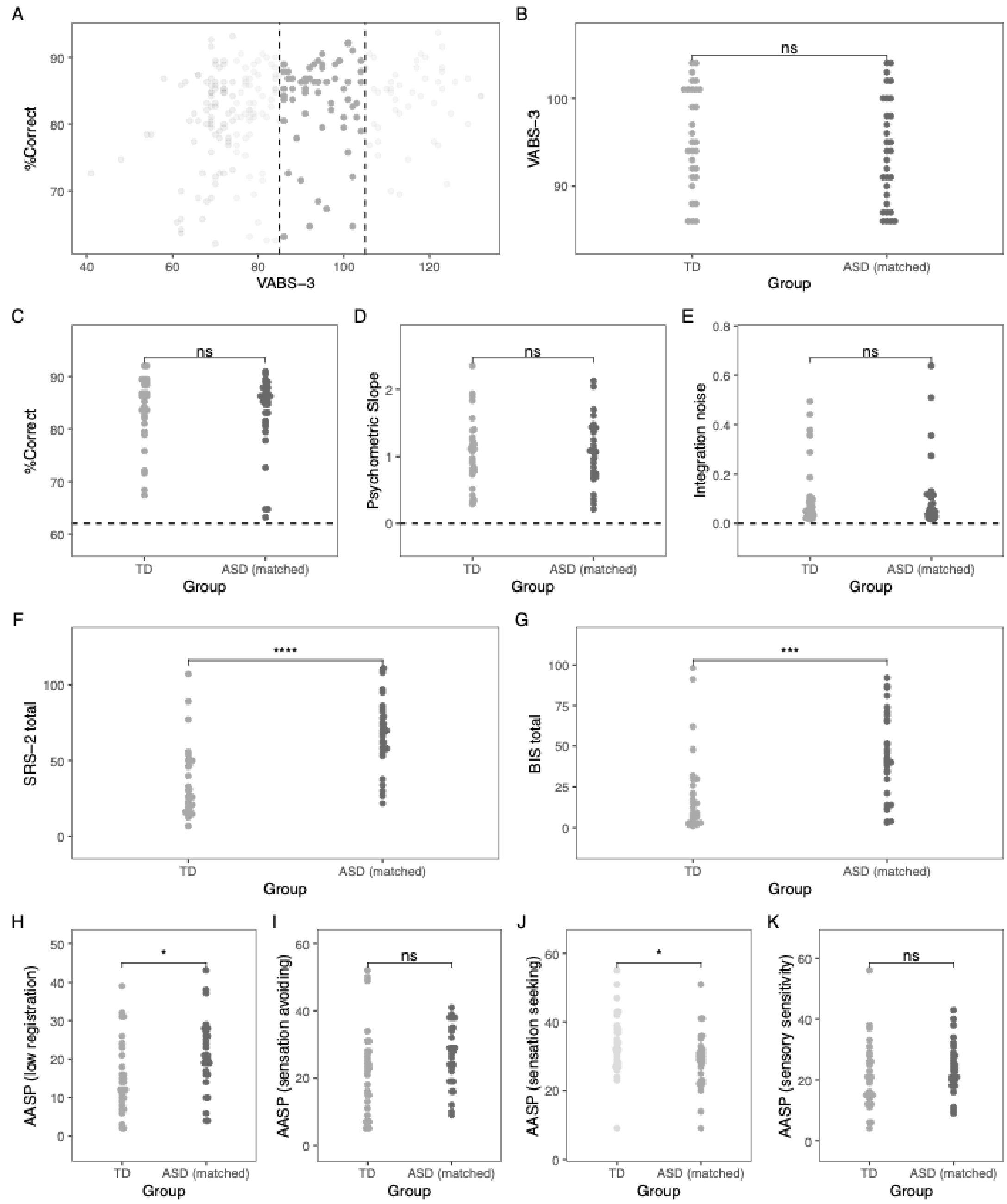

Figure S9. Comparing ASD and TD matched groups with similar VABS-3 scores. A. The analysis included 30 typically developing (TD) participants and 31 ASD participants matched on adaptive functioning. VABS-3 scores were restricted to the range 85–105, with inclusion limits indicated by dashed lines. **B.** Within this subsample, TD and ASD participants showed comparable VABS-3 scores. **C–E.** No significant group differences were observed in game-derived measures, including percentage correct **(C),** psychometric slope **(D),** and integration noise **(E). F–K.** Group differences emerged in survey-based measures: SRS-2 (F; $t(58.76) = -5.55$, $p < 0.001$), BIS (**G**; $t(58.99) = -3.66$, $p < 0.001$), and AASP subdomains—low registration (**H**; $t(58.81) = -2.50$, $p < 0.05$) and sensation seeking (**J**; $t(57.74) = 2.25$, $p < 0.05$), with no significant differences in sensation avoiding (**I**) or sensory sensitivity (**K**).
